# Hyperbaric Oxygen Potentiates Doxil Antitumor Efficacy by Promoting Tumor Penetration and Sensitizing Cancer Cells

**DOI:** 10.1002/advs.201700859

**Published:** 2018-06-25

**Authors:** Xian Wu, Yanhong Zhu, Wei Huang, Jingqiu Li, Bixiang Zhang, Zifu Li, Xiangliang Yang

**Affiliations:** ^1^ National Engineering Research Center for Nanomedicine College of Life Science and Technology Huazhong University of Science and Technology Wuhan 430074 P. R. China; ^2^ Huazhong University of Science and Technology Tongji Med College Tongji Hospital Hepat Surg Ctr, 1095 Jiefang Ave Wuhan 430030 P. R. China; ^3^ Hubei Key Laboratory of Bioinorganic Chemistry and Materia Medica Huazhong University of Science and Technology Wuhan 430074 P. R. China; ^4^ Wuhan Institute of Biotechnology High Tech Road 666 East Lake High Tech Zone Wuhan 430040 P. R. China

**Keywords:** cancer cell sensitization, chemo/HBO combination therapy, Doxil, hyperbaric oxygen, tumor penetration

## Abstract

Hypoxia is a fundamental hallmark of solid tumors and helps contribute to chemotherapy resistance. Hyperbaric oxygen (HBO) therapy can overcome tumor hypoxia and promote chemotherapy antitumor efficacy; however, the simultaneous administration of some conventional chemotherapies, including doxorubicin (DOX), with HBO is considered an absolute contraindication. Here, DOX‐loaded liposome (Doxil) is coadministered with HBO to assess the safety and efficacy of this combination treatment. By overcoming tumor hypoxia, HBO not only improves Doxil tumor penetration by decreasing the collagen deposition but also sensitizes tumor cells to Doxil. As a result, the combination treatment synergistically inhibits H22 tumor growth, with a tumor inhibition rate of 91.5%. The combination of HBO with Doxil shows neither extra side effects nor promotion of tumor metastasis. These results collectively reveal that the combination of HBO with Doxil is an effective and safe treatment modality. As both HBO and Doxil are routinely used, their combination could quickly translate to clinical trials for patients with hypoxic solid tumors.

## 1 Introduction

Hypoxia plays a central role in tumor biology,^1^ as it not only fuels tumor development, progression, and metastasis, but also induces chemotherapy resistance both in pharmacokinetics and pharmacodynamics.^2^ Hypoxia upregulates hypoxia induced factor 1α (HIF‐1α)^3^ and collagen,^4^ which builds up a dense extracellular matrix (ECM) and severely limits drug delivery efficiency and antitumor efficacy.^5^ Moreover, tumor cells under hypoxic condition are often caught in an inactive state and are insensitive to chemotherapeutic agents.^6^ Therefore, overcoming hypoxia can significantly potentiate chemotherapy. To that end, various systems aiming at raising oxygen tension at hypoxic solid tumors have been urgently pursued.^7^ Most of these studies, however, are still at the bench stage and far from being used as bedside applications.

Hyperbaric oxygen (HBO) therapy can overcome tumor hypoxia and has been routinely utilized in clinics for many years.^8^ Operating at elevated pressure, typically 2–3 atmosphere absolute (ATA), HBO increases the oxygen concentration in the plasma and therefore facilitates oxygen delivery directly. HBO is considered a safe clinical treatment and has been used for ischemia, acute carbon monoxide poisoning, nonhealing wounds, and late radiation injury.^9^ As an effective approach in elevating oxygen content, HBO has already been combined with radiotherapy and photodynamic therapy for hypoxic solid tumor treatment.^10^ However, HBO is seldom combined with chemotherapy, especially drugs which execute antitumor effect through reactive oxygen species (ROS), such as DOX, because HBO may exasperate the side effects of these drugs.[[qv: 8a]] Thus, the simultaneous administration of DOX with HBO is considered an absolute contraindication.

Through enhanced permeability and retention (EPR) effects, nanomedicines selectively accumulate at the malignant cancerous tissues to mitigate the adverse reactions induced by conventional chemotherapies, both in laboratory studies and clinical settings.^11^ Dozens of nanomedicines, such as Doxil, have been approved by the Food and Drug Administration (FDA).^12^ However, the nanoscale size of these nanomedicines critically restricts their penetration in the dense ECM of hypoxic solid tumors, delivery efficiency, and antitumor efficacy.^13^ To bolster nanomedicine tumor penetration and antitumor efficacy, two distinctive approaches have been investigated. One approach focuses on the design of sophisticated and smart nano‐drug delivery systems that are often difficult to scale up and translate.^14^ The other approach primes the tumor microenvironment (TME) to cater for the subsequent drug delivery.^15^ For example, in overriding the hypoxic condition in solid tumors, HBO can effectively modulate TME to decrease collagen content and interstitial fluid pressure (IFP).^16^ Nonetheless, to the best of our knowledge, leveraging HBO to boost nanomedicine tumor penetration and improve antitumor efficacy has not been previously reported.

**Scheme 1:**
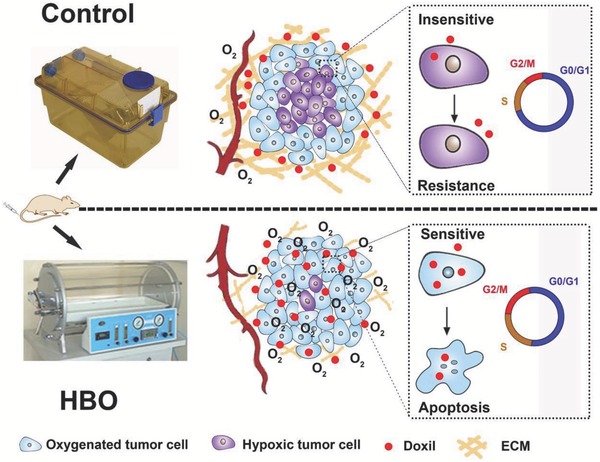
Schematic illustration of combination therapy based on HBO and Doxil. By mitigating tumor hypoxia, HBO decreases collagen content, resulting in the enhanced penetration of Doxil into the tumor. HBO also notably interrupts cancerous cell cycle arrest, rendering hypoxic tumor cells more sensitive to Doxil.

Here, for the first time, we combined two FDA approved therapies, HBO and Doxil, to synergistically potentiate Doxil antitumor efficacy. By overcoming tumor hypoxia, HBO not only decreases collagen deposition and promotes Doxil penetration into the tumor but also interrupts cancerous cell cycle arrest, rendering hypoxic tumor cells more sensitive to Doxil (see **Scheme** **1**). Moreover, Doxil significantly mitigates the cardiotoxicity of DOX under HBO. Collectively, these results suggest that the combination of HBO with Doxil, and likely many other nanomedicines, is an effective and safe treatment modality for hypoxic solid tumors and could facilely translate to clinical trials.

## 2 Results and Discussion

According to hospital practice, HBO operates between 2–3 ATA for 2 h, undergoing pressurization for 15 min and depressurization for another 15 min, and reaching 2.5 ATA with >97% oxygen atmosphere for 1.5 h. However, this process is believed to damage the stability of Doxil. To assess this concern, we first examined the stability of Doxil under HBO. We measured the size, zeta potential, and release behavior of Doxil with HBO therapy. Each HBO therapy treatment conducted in this study lasted 2 h. No significant change was observed after Doxil was exposed to the HBO treatment. The diameter of Doxil is consistently around 80 nm before and after HBO treatment, the zeta potential is always slightly negative charged (around −3 mV), and the DOX release curves are perfectly overlapped (Figures S1 and S2, Supporting Information). These results demonstrate that HBO exerts a negligible effect on the stability of Doxil.

### 2.1 HBO Therapy Alleviates Tumor Hypoxia

**Figure 1:**
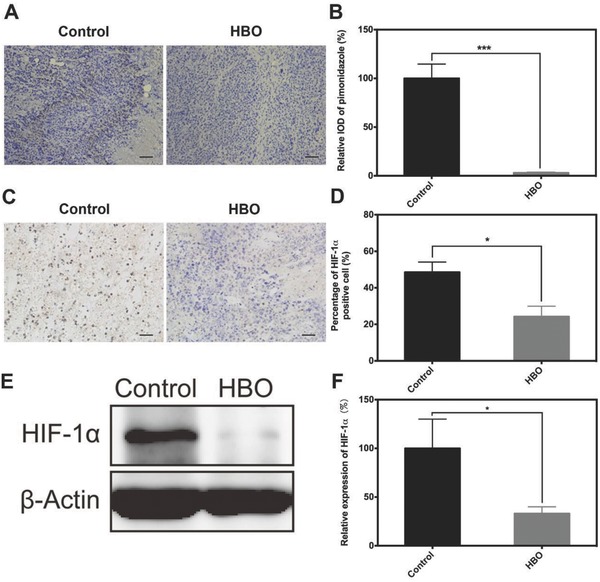
HBO therapy overcomes tumor hypoxic microenvironment. Immunohistochemistry analysis of A) pimonidazole and C) HIF‐1α. E) The expression of HIF‐1α in tumor tissue. B,D,F) Semi‐quantitative analysis of (A), (C), and (E), respectively. The scale bar is 50 µm in (A) and (C). Data as mean ± S.E. (*n* = 3). *P < 0.05, ***P < 0.001.

Previous studies have reported several methods for alleviating tumor hypoxia to enhance therapeutic efficacy.^17^ However, most of these methods are still at the laboratory stage and have yet to be translated for clinical use. By stark contrast, HBO treatment has been widely used for decades in clinics and has become an effective method for oxygenation.^9^ Various types of cancers have been investigated as hypoxic tumors, including pancreatic cancer,^18^, ^19^ liver cancer,[[qv: 7b,20]] and breast cancer.[[qv: 7d,18]] Here, liver cancer is selected for two considerations. First, liver cancer is widely used as a typical tumor model with rich ECM and severe hypoxia tumor microenvironment.^21^ Second, for the purpose of clinical translation, we applied clinical‐approved nanomedicine, Doxil, to combine with HBO. Both DOX and Doxil are widely used for liver cancer. Hence, liver cancer cells, H22, Bel‐7402, and HepG2, are used in this study. To confirm that HBO therapy oxygenates hypoxic tumor tissues, we used a hypoxia‐probe, pimonidazole, to detect the hypoxic area of H22 subcutaneous tumor tissue after one HBO treatment. Immediately after the HBO treatment, pimonidazole was administrated through an intravenous injection. After 90 min, the mice were sacrificed and tumor tissues were harvested. Then, anti‐pimonidazole mouse IgG_1_ monoclonal antibody (MAb1) was used for immunochemical detection of hypoxia in tumor tissue according to manufacturer instructions. The immunohistochemistry of pimonidazole after HBO treatment is shown in **Figure** **1**A. The hypoxic area uncovered by the relative intensity of optical density (IOD) of pimonidazole significantly decreased by 90% (*P* < 0.001) after a single HBO treatment (Figure 1B).

To further validate that the oxygenation of hypoxic tumor could indeed exert a meaningful biological effect, we quantified two important indicators in tissue hypoxia and TME, HIF‐1α, and its downstream target vascular endothelial growth factor (VEGF), after three HBO therapies.^22^ The percentage of HIF‐1α positive cells in the HBO group was 24.4 ± 5.7% and was significantly lower than that of the control group (48.5 ± 5.6%) (Figure 1C,D). A 67% reduction in the expression of HIF‐1α was also observed with the western blotting (Figure 1E,F). Collectively, these findings show that HBO effectively modulates hypoxia. As a downstream target of HIF‐1α, the expression of VEGF should be mediated by HIF‐1α. Accordingly, there was an 85% reduction in expression of VEGF observed after HBO therapy, revealing that the expression of HIF‐1α is indeed decreased (Figure S3, Supporting Information).

Pimonidazole, HIF‐1α, and VEGF are important prognostic markers of hypoxia in human cancer^23^ and are widely used for detecting hypoxia modulation.^22^, ^24^ In this study, each of these three markers showed that HBO overcame tumor hypoxia. Similar conclusions have been drawn by using magnetic resonance imaging and oxygen electrodes.^25^ Taken together, Figure 1 exhibits that HBO therapy overcomes tumor hypoxia and downregulates HIF‐1α expression, which can benefit cancer treatment.

### 2.2 HBO Therapy Promotes Penetration of Doxil in Tumor Tissue by Modulating Tumor ECM

Deep penetration of therapeutic drugs, especially for nanotherapeutics, is a bottleneck for efficient drug delivery and potent cancer chemotherapy.^26^ Because of the dense ECM and high IFP, the penetration distance of most anticancer drugs is only 3–5 cell diameters, typically less than 200 µm.^27^ Few drugs are able to reach the tumor tissue core, which is often hypoxic, and kill those aggressive tumor cells. Tumor ECM consists of a highly interconnected network of collagen fibrils that become the major barrier for interstitial transportation of drugs. Thus, lowering the content of collagen fibrils would facilitate drug deep penetration.[[qv: 15c]] Previous studies showed clear link between tumor hypoxia and the deposition of collagen fibrils: Higgins et al. found that hypoxia‐induced connective tissue growth factor (CTGF) is mainly mediated by HIF‐1 α^28^ and similar results were revealed in renal system^29^ and in tumors.^30^ And CTGF acts as a critical regulator on collagen deposition in tumor tissue. For instance, Zhu et al. discovered that overexpression of CTGF in human mammary epithelial cells expressing LT, hTERT and H‐rasV12 tumor leads to an enlarged ratio of fibrous area as well as increased fibril content.^31^ Chauhan et al. utilized losartan to inhibit the expression of CTGF in order to remodulate tumor ECM and achieve better drug delivery.^18^ Our data (Figure 1E,F) have demonstrated that HBO reduces the amount of HIF‐1α significantly, implying HBO might be able to inhibit collagen fibers deposition by interrupting HIF‐1α/CTGF/collagen pathway. Therefore, the effects of HBO therapy on tumor ECM were studied next.

**Figure 2:**
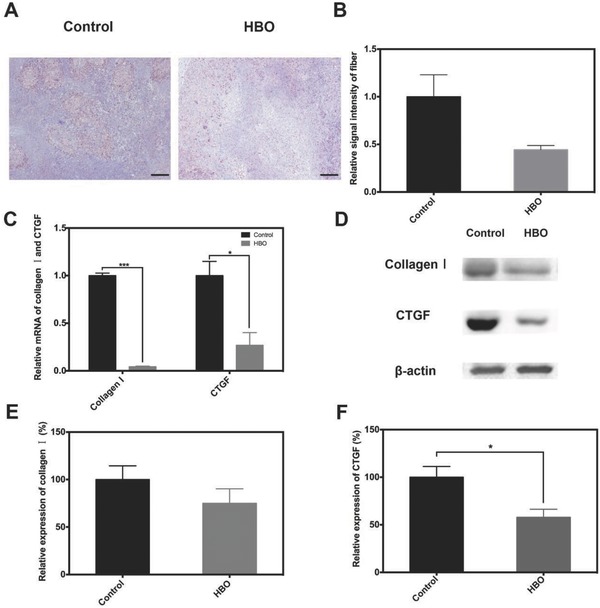
HBO therapy decreases the collagen fibrils in tumor tissue. H22 subcutaneous tumor‐bearing mice were either treated with HBO therapy (HBO) or without HBO therapy (control) once a day for 3 d. A) Masson staining of tumor tissue. B) Semi‐quantitative analysis of (A). C) Relative mRNA of collagen I and CTGF in tumor tissue. D) The protein expression of collagen I and CTGF in tumor tissue. E,F) Semi‐quantitative analysis of (D). Scale bar is 50 µm in (A). Data as mean ± S.E. (*n* = 5). *P < 0.05, ***P < 0.001.

Tumor‐bearing mice were administrated HBO therapy daily for three successive days. 24 h after the last therapy, the mice were sacrificed and tumor tissues were harvested. Masson's trichrome stain was carried out according to the standard protocol. The images of the Masson's trichrome stain reveal that the deposition of fibrils decreases after HBO therapy, as shown in **Figure** **2**A. The semi‐quantitative analysis exhibited a 44% reduction of fibril content in HBO group compared to that of control group (Figure 2B). To obtain more insights on the pathway which mediated collagen deposition, we quantified the expression of collagen I, one of the major components of collagen fibrils, and CTGF, which is the up‐stream factor regulating the deposition of collagen I. The results of the real‐time polymerase chain reaction, (RT‐qPCR), test are shown in Figure 2C. A significant down‐regulation of transcription for both CTGF and collagen I after HBO therapy can be clearly seen. Gene transcription of CTGF and collagen I decreased by 73% and 95%, respectively. We further quantified protein content of CTGF and collagen I through western blotting, as shown in Figure 2D. The semi‐quantitative analysis of the western blotting (Figure 2E,F) showed that the expressions of CTGF and collagen I in HBO group were 58% and 75% of that in control group, respectively. These findings are highly consistent with the RT‐qPCR data.

Taken together, these results show that HBO therapy reduced the deposition of collagen in tumor ECM. These results are aligned with published work which has shown that the number of collagen fibril per µm^2^ in tumor tissue decreased 66.7% after HBO therapy.^16^ Combined with the immunohistochemistry and western blotting results of HIF‐1α, the results showed in Figure 2 demonstrate that HBO therapy decreases collagen fibril deposition through the regulation of HIF‐1α/CTGF/collagen I pathway.

**Figure 3:**
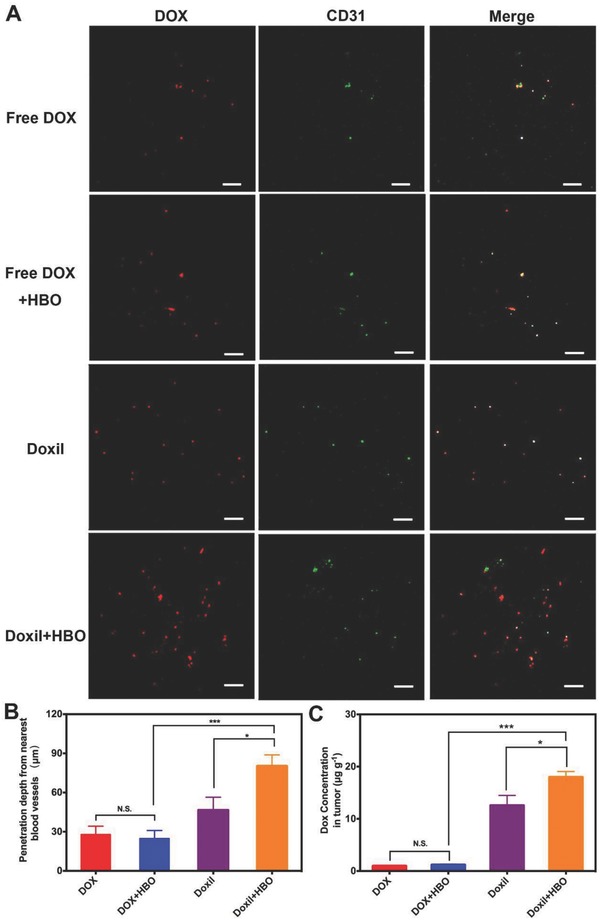
In vivo drug penetration and tumor DOX accumulation. A) In vivo penetration of DOX into the tumors of H22‐bearing mice after intravenous injection of free DOX/Doxil at DOX dosage of 7 mg kg^−1^ with and without HBO therapy. The frozen tumor sections were observed at 24 h after injection using confocal microscopy. The blood vessels were stained by FITC‐CD31 antibody. The scale bar is 200 µm. B) The tumor penetration distance of DOX from the nearest blood vessel was determined with the simulated scatter diagrams method. C) The concentration of DOX in tumor tissue in H22‐bearing mice after intravenous injection of free DOX/Doxil at DOX dosage of 7 mg kg^−1^ with and without HBO therapy for 24 h. Data as mean ± S.E. (*n* = 5). *P < 0.05, ***P < 0.001. N.S. as not significant.

Decreased collagen I deposition in tumor ECM is expected to boost Doxil deep penetration and accumulation in tumor tissue.[[qv: 5a,15c,32]] Fluorescence immunohistochemistry was applied to study the penetration of Doxil and the absolute DOX concentration was determined by extracting DOX from tumor tissues. Tumor‐bearing mice were divided into four groups: DOX, Doxil, DOX+HBO, and Doxil+HBO (the details of these groups are provided in the Supporting Information). The frozen slides of each treatment group are shown in **Figure** **3**A (also see Figure S4, Supporting Information). The fluorescent intensity within the Doxil+HBO group is significantly higher than that of any other group, indicating an elevated DOX content in tumor tissue in the Doxil+HBO group. Notably, most of the red (DOX) fluorescence in the DOX, DOX+HBO, and Doxil groups is colocated with the fluorescence of blood vessels, which are stained with FITC‐CD31 antibody and exhibit green fluorescence (Figure 3A). This suggests that these groups have poor drug penetration. However, in striking contrast, the red fluorescence is scattered within the entire tumor section in Doxil+HBO, implying the enhanced penetration of Doxil.

Based on five confocal images, penetration distances were calculated using simulated scatter diagrams^33^ (Figure 3B, see the Supporting Information for detailed description). Doxil+HBO exhibits the highest penetration distance, which is 80.12 ± 8.72 µm, ≈2.87, 3.23, and 1.73 times of that in DOX, DOX+HBO, and Doxil, respectively. It should also be noted that nearly the same penetration distances are achieved for DOX with or without HBO; whereas, Doxil penetrates 1.73 times deeper with HBO than without. This observation indicates that HBO boosts Doxil tumor penetration but offers little help to DOX, justifying the advantage to combine HBO with Doxil. The amount of DOX accumulated within tumors was further quantified by extracting DOX from tumor tissues and determining the DOX concentration with a fluorescence spectrophotometer. The amount of DOX per gram of tumor tissue was 0.93, 1.15, 12.54, and 17.95 µg for the DOX, DOX+HBO, Doxil, and Doxil+HBO treatments, respectively. Because of its optimum tumor penetration,[[qv: 14b,34]] the highest DOX concentration was observed in Doxil+HBO (Figure 3C). A 41% increase of DOX concentration in the tumor is observed after the HBO treatment with Doxil; however, the HBO treatment has little influence on free DOX.

Unlike previous reports suggesting that HBO therapy increases the content of small molecular drugs in tumor tissues,^35^ our results demonstrate that HBO therapy increases penetration distance and tumor accumulation exclusively for Doxil but not for free DOX. This can likely be ascribed to the fact that free DOX does not circulate in the body long enough for extravasation, penetration, and accumulation. DOX has a half‐life time *t*_1/2_ of 8.7 h, whereas Doxil has a *t*_1/2_ of 45.2 h.^36^ After entering systemic circulation, free DOX is rapidly cleared out through the kidney (Figure S5, Supporting Information). Additionally, even though HBO therapy decreases tumor fibril deposition (Figure 2A) and facilitates the deep penetration of free DOX into tumor tissue, free DOX does not retain at the tumor site. Being a molecular antitumor agent, DOX is easily drained out from tumor tissue. However, as a nanomedicine, Doxil has a size around 80 nm and can remain at the tumor through the EPR effect. Under these circumstances, HBO therapy is more effective as an adjuvant method with nanotherapeutics than with small molecular anticancer chemotherapies (Figure 3).

These findings (Figures 2 and 3) clearly demonstrate that HBO opens up the dense ECM in hypoxic solid tumors and selectively benefits Doxil rather than DOX. To the best of our knowledge, this is the first report in which HBO therapy acts as a facilitative therapy for deep penetration of nanotherapeutics. A variety of methods have also been developed to bolster penetration of nanotherapeutics,^37^ and many of these focus on degradation of tumor ECM by delivering small molecular drugs or enzymes. Compared with these methods, HBO uses oxygen as a drug to oxygenate hypoxic tumors and modulate ECM. Oxygen has the advantage of higher delivery and penetration efficiencies under HBO situations. Moreover, the drugs and enzymes used to degrade ECM in those methods have safety concerns that must be alleviated with clinical trials, whereas HBO has been used for many years with a proven safety profile. Collectively, our results show that HBO therapy reinforces Doxil penetration and enhances DOX accumulation at tumor tissue. These actions are beneficial for in vivo antitumor efficacy.

### 2.3 HBO Therapy Sensitizes Tumor Cells to Chemotherapeutics by Modulating Cell Cycle Arrest and Intracellular DOX Concentration

**Figure 4:**
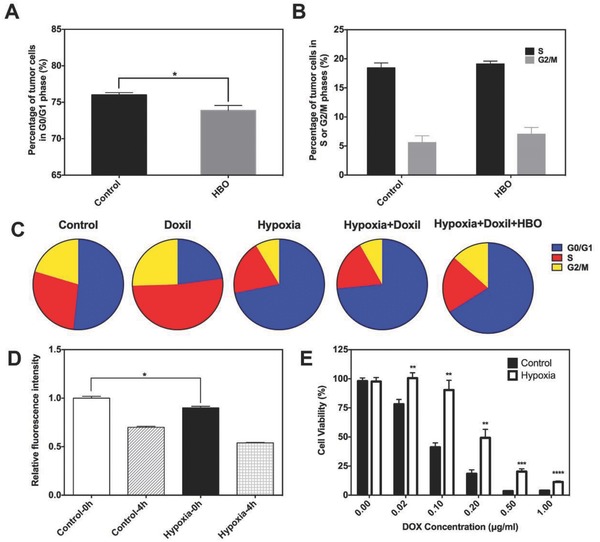
HBO sensitizing cancer cells to DOX. A,B) In vivo cell cycle analysis. C) In vitro cell cycle analysis. D) Cell uptake and efflux study. The relative fluorescence intensity of DOX after H22 cells was treated with 5 µg mL^−1^ DOX in serum‐free medium for 2 h. Cells were then washed with phosphate buffer saline (PBS) three times and then the relative fluorescence intensity of DOX within cells was determined by flow cytometry (shown as Control‐0 h and Hypoxia‐0 h). H22 cells were then reincubated for another 4 h and washed again. The relative fluorescence intensity of DOX inside cells was again determined by flow cytometry (shown as Control‐4 h and Hypoxia‐4 h). E) The cell viability of H22 cells treated with different concentrations of DOX at 48 h by CCK‐8 assay. Cells were preincubated under hypoxia (1% O_2_) for 24 h in hypoxia group. Data as mean ± S.E. (*n* = 3). *P < 0.05, **P < 0.005, ***P < 0.001, ****P < 0.0001.

As the fundamental hallmark of most solid tumors, hypoxia has been found to be responsible for chemotherapy resistance.^38^ Hypoxia‐induced cell cycle arrest is one of the main contributors for chemotherapy resistance and has been studied for many years. Because most anticancer drugs are effective against rapidly dividing cells, hypoxia‐induced cell cycle arrest renders cells insensitive to antitumor agents.^39^ To confirm that alleviation of hypoxia could modulate hypoxia‐induced cell cycle arrest, the cell cycle of cells from tumor tissue after HBO were studied. Tumor‐bearing mice (Bel‐7402 in Balb/c‐nude mice; see the Supporting Information) were treated with HBO therapy daily for 3 days. 22 h after the last therapy, tumor tissue was harvested and immediately treated with collagenase to prepare a single cell suspension. Cells were then fixed and stained with propidium iodide (PI) according to standard protocol. The percentage of cells in G0/G1 phase decreased from 76% to 73% in tumor tissues after HBO therapy, **Figure** **4**A (representative raw data are shown in Figure S6, Supporting Information). Although only 3% more cells entered S or G2/M phase, the difference is statistically significant. There is no significant difference in the percentage of cells in S or G2/M phase between the control group and HBO group (Figure 4B). These data verify that HBO therapy can overcome hypoxia‐induced cell cycle arrest in vivo.

To gain more insights about the effects of HBO therapy on modulating cell cycle, which sensitizes tumor cells to Doxil, the Bel‐7402 cell line was studied under different conditions in vitro. In vitro hypoxic incubation of tumor cells is used to simulate the in vivo hypoxic microenvironment. Cells were divided into five groups, and the details of each group are specified in Table S2 (Supporting Information). After different treatments, cells were fixed with 70% ethanol overnight. PI was then applied to stain the DNA according to the standard protocol. Figure 4C shows in vitro cell cycle results and Table S3 (Supporting Information) summarizes the detailed statistics (representative raw data are shown in Figure S7, Supporting Information). Compared to the control (control group, normal condition, ≈52% cells in G0/G1 phase), about 70% of cells are arrested in G0/G1 phase due to hypoxia (hypoxia group). This cell arrest in hypoxic condition is consistent with previous studies.[[qv: 39b,40]] As DOX causes DNA damage during DNA replication, cells in the Doxil group are mainly in S phase (≈51%) and apoptosis will be the destiny to these cells. Interestingly, when cells are incubated with Doxil under hypoxic conditions (hypoxia+Doxil group), about 73% of cells remain in G0/G1 phase rather than in S phase. This pattern of cell cycle is similar to that of hypoxia group. This seemingly implies that cells are arrested in G0/G1 phase and do not response to Doxil under hypoxia. Next, we administered one single HBO therapy to hypoxic cells immediately after adding Doxil to the medium. After a 2 h HBO therapy, cells were further cultured in hypoxic condition for another 22 h (designated as hypoxia+Doxil+HBO, Supporting Information). Only about 60% of cells are arrested in G0/G1 phase compared to 73% in the hypoxia+Doxil group, indicating that HBO therapy “wakes up” about 13% cells. This difference substantiates that HBO therapy sensitizes tumor cells to Doxil in vitro by combating hypoxia‐induced G0/G1 arrest.

Another important parameter contributing to cancer cell chemotherapy insensitivity is inadequate intracellular drug concentration. We measured intracellular drug content under different conditions by flow cytometry. Again, in vitro hypoxic incubation of tumor cells is used to simulate the in vivo hypoxic microenvironment. Briefly, cells were preincubated under normoxic (20% O_2_) or hypoxic conditions (1% O_2_) for 24 h followed by incubating with DOX (5 µg mL^−1^). After incubating with DOX for 2 h (time for cells to uptake DOX), the fluorescence intensity within cells was recorded with flow cytometry, and cells were then immediately transferred to DOX‐free medium for another 4 h (time for cells to excrete DOX) under normoxia or hypoxia. Subsequently, the fluorescence intensity within cells was measured again. Hypoxia significantly reduced DOX uptake of H22 cells by 11% (hypoxia‐0 h vs control‐0 h, *P* < 0.05) for the first 2 h (Figure 4D). After incubating the cells for 4 h to allow DOX excretion, the fluorescence intensity decreased in both groups as a result of cellular efflux. However, cells under hypoxia excreted more DOX than cells under normoxia (hypoxia‐4 h vs control‐4 h, *P* < 0.05). Fluorescence intensity was reduced by 30.3% in the control group and 40.2% in the hypoxia group. These results indicate that cells under hypoxia uptake less (around 11%) and excrete more (around 10%) DOX when compared with cells under normoxia (*P* < 0.05), together contributing to a relatively lower intracellular DOX concentration under hypoxia.

After confirming that HBO therapy could overcome G0/G1 cell cycle arrest and increase intracellular drug concentration, we tested whether the elevated oxygen pressure can sensitize tumor cells to DOX. A CCK‐8 kit was used to evaluate the viability of H22 cell treated with DOX under normoxia and hypoxia. In vitro hypoxic incubation was used to simulate the oxygen condition of cells under tumor hypoxia. Cells were preincubated under normoxia or hypoxia for 24 h. DOX was then added with fetal bovine serum (FBS)‐free medium and cells were incubated under both normoxia and hypoxia for 24 or 48 h. The viability of the cells was tested at indicated time points. We observed that cells under hypoxia were less sensitive to DOX than cells under normoxia both at 24 (Figure S8, Supporting Information) and 48 h (Figure 4E). For example, when the DOX concentration is 0.1 µg mL^−1^, more than 90% of the hypoxic cancer cell survives, whereas less than 40% of cancer cell remains alive under normoxia (*P* < 0.01). When the DOX concentration is raised to 0.5 µg mL^−1^, 20% hypoxic cancer cells still survive, but almost all cells are killed under normoxia. The IC_50_ of DOX to H22 cells under normal condition is 0.065 µg mL^−1^ for 48 h. However, when cells are under hypoxia condition, the IC_50_ of DOX increases to 0.23 µg mL^−1^ for 48 h. Cells are roughly 3.5 times more difficult to be killed by DOX under hypoxia, emphasizing the importance of cancer cell sensitization by HBO in cancer treatment.

Reversing chemoresistance via enhancing oxygenation of a tumor is a current research focus of many research groups. Our data show that IC_50_ of DOX rises 3.5 times when cells are under hypoxic condition. Similar results have also been reported that hypoxia decreased cytotoxicity of several antitumor agents.[[qv: 6b,39a]] Many mechanisms have been proposed to account for the poor response of tumor cells to antitumor agents under hypoxia; however, for this study, we focused on intracellular drug concentration and cell cycle arrest. We found that cells under hypoxic conditions uptake less and excrete more DOX, contributing to lower intracellular DOX concentration. Other studies provide supporting evidence with reasonable explanations for our findings. Hypoxia has been discovered to upregulate the expressions of P‐gp and multi‐drug resistance (MDR), which helps excrete drugs out of the tumor cells.[[qv: 7d,41]] Alleviating hypoxia via HBO therapy could downregulate the expression of HIF‐1α, P‐gp, and MDR, and therefore increases intracellular drug content. Another possible mechanism for hypoxia‐induced insensitivity of tumor cells to antitumor agents is cell cycle arrest. Previous studies have also proven that hypoxia‐induced cell cycle arrest impaired cytotoxicity of 5‐fluorouracil,^40^ antifolate,^42^ DOX, and methotrexate.[[qv: 39a]] Using HBO therapy to combat hypoxia‐induced cell cycle arrest has already been reported. Kalns and Piepmeier showed that HBO therapy modulates cell cycle arrest in a pressure‐dependent manner.^43^ Zhang et al. pointed out that HBO could induce tumor cells to accumulate at the S phase, a cell phase that is particularly sensitive to chemotherapy and radiotherapy.^44^ Similarly, in our in vitro experiment, we find that when tumor cells are under normal conditions, administrating Doxil causes DNA damage and arrests tumor cells in S phase of cell cycle (which ultimately leads to cell apoptosis). However, because of tumor hypoxic microenvironment, most of cells are arrested in G0/G1 phase and do not response to Doxil. After HBO treatment, the hypoxia is alleviated. As a consequence, tumor cells escape from G0/G1 cell cycle arrest and become sensitive to Doxil. In this way, HBO led to the sensitization of tumor cells to Doxil.

However, all these previous studies and our work to this point have been carried out in in vitro conditions. Whether HBO therapy can modulate cell cycle arrest in vivo is still unknown. Given this background, for the first time we reveal that utilizing HBO therapy can decrease the percentage of cells arrested in G0/G1 phase in a mouse model (Figure 4A). This observation implies that HBO therapy may reinforce chemotherapy antitumor efficacy in vivo. Moreover, in addition to the two mechanisms previously discussed, ROS generation^45^ is known to play a vital role in cancer cell sensitization. This mechanism has been well studied and may contribute to the results shown in Figure 4. Taken together, Figure 4 illustrates that hypoxia‐induced DOX resistance can be effectively overridden by HBO therapy via interrupting G0/G1 cell cycle arrest and elevating intracellular drug concentration.

### 2.4 The Combination of HBO Therapy and Doxil Promotes Antitumor Efficacy

We have thus confirmed that HBO therapy overcomes tumor hypoxia (Figure 1), decreases collagen deposition in tumor ECM (Figure 2), boosts Doxil tumor penetration (Figure 3), potentiates Doxil tumor accumulation (Figure 3), and sensitizes tumor cells to DOX (Figure 4). Encouraged by these promising results, we further investigated the in vivo antitumor activity on a subcutaneously transplanted H22‐tumor mouse model.

**Figure 5:**
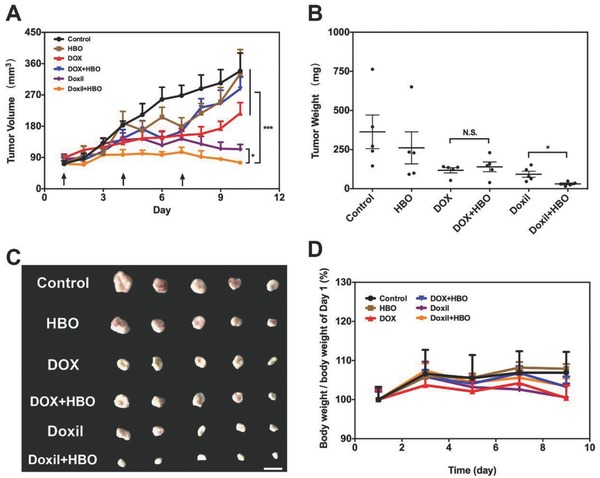
In vivo antitumor effects. A) Tumor growth inhibition profiles in H22‐bearing mice after intravenous injection of free DOX and DOX‐loaded liposome at DOX dosage of 4 mg kg^−1^ with and without HBO therapy. For control and HBO, saline was used. B) Tumor weight at the end of tumor growth inhibition studies. C) Representative photos of H22 tumor tissue at the end of tumor growth inhibition studies. The scale bar is 10 mm. D) Body weight change profiles during the tumor growth inhibition studies. Data as mean ± S.E. (*n* = 5). *P < 0.05, ***P < 0.001. N.S. as not significant.

Tumor‐bearing mice were randomly separated into six groups: Control, HBO, DOX, DOX+HBO, Doxil, and Doxil+HBO. Details of the different groups are showed in Table S4 (Supporting Information). The change of tumor volume and body weight was monitored daily during the experiment. **Figure** **5**A shows the tumor‐inhibition profile based on tumor volume. Tumors in the control group grew vigorously during the experiment and the average volume reached about 300 mm^3^. HBO therapy alone did not affect the growth of tumors, and there is no significant difference compared with control (*P* > 0.05). Groups receiving free DOX (both DOX and DOX+HBO groups) showed a modest tumor‐inhibition effect in the early stage, and the volume increased quickly afterward. Doxil alone exhibited significant antitumor effect from day 5 onward and the mean volume at the end of the experiment was around 110 mm^3^. The combination therapy of Doxil and HBO (Doxil+HBO) achieved the best tumor‐inhibition effect, with mean volume around 75 mm^3^, which is significantly smaller than that of using Doxil alone (*P* < 0.05).

Two days after the final administration, the mice were sacrificed and tumor tissues were excised and weighted. Figure 5B exhibits the final tumor weight in different groups. The tumor inhibition rate based on the tumor weight is 61.6% for DOX+HBO, which is close to DOX (67.7%, *P* > 0.05 compared with DOX+HBO), and 91.5% for Doxil+HBO, which is significantly higher than that of Doxil (74.7%, *P* < 0.05 compared with Doxil+HBO), suggesting a strong synergistic effect between Doxil and HBO. And these results are highly consistent with data of tumor penetration and accumulation (Figure 3). The excised tumors show a similar trend as tumor weight (Figure 5B,C). Body weight of mice in the different treatment groups is shown in Figure 5D. No significant differences among the groups can be observed, indicating that there are no severe side effects. It is worth noting that HBO therapy alone exerts negligible effects (neither stimulation nor inhibition) on tumor growth after investigating tumor volume, tumor weight, and images. These data corroborate that HBO and Doxil synergistically inhibit tumor growth, without obvious side effects, and that HBO therapy alone does not stimulate tumor growth.

It has been previously reported that HBO therapy alone can exert inhibitory effects against hypoxic solid tumors. However, this is tumor dependent. In our liver cancer model, we do not observe evident inhibitory effect, which suggests that H22 tumors, similar to C3HBA murine^46^ tumors and squamous cell cancer (SCC) xenografts,^47^ hardly respond to HBO therapy. We also tested whether HBO therapy alone can inhibit tumor growth in vitro (Figure S9, Supporting Information). Within all the cell lines we tested, five consecutive HBO therapies significantly inhibit cell viability for each, except the LM3 cell line. These results indicate that the inhibitory effect of HBO therapy is dependent on the tumor‐type and can be drastically different from in vitro to in vivo.

Unlike previous reports suggesting that HBO therapy enhances the therapeutic effect of molecular antitumor agents,^35^ there is no significant difference in tumor volume and tumor weight between DOX and DOX+HBO in our experiment. However, the Doxil+HBO group does significantly differ from the Doxil group in terms of tumor volume and tumor weight. These results can be ascribed to the enhanced Doxil penetration and tumor accumulation (Figure 3), emphasizing that HBO therapy is more effective as an adjuvant method with nanotherapeutics than with molecular antitumor agents. The in vivo antitumor data justify the use of the combination therapies based on HBO and Doxil rather than with free DOX. The in vivo antitumor result demonstrates that the combination therapies based on Doxil and HBO synergistically potentiates Doxil antitumor effect.

### 2.5 The Combination of HBO Therapy and Doxil Shows Modest Side Effects and No Promotion for Tumor Proliferation and Metastasis

**Figure 6:**
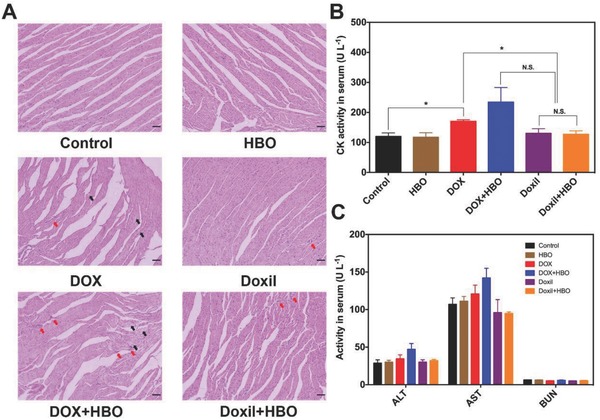
Side effect analysis. A) H&E analysis of heart tissue at the end of tumor growth inhibition studies. Red arrow represents neutrophil accumulation and black arrow represents myocardial necrosis. The scale bar is 50 µm. B) CK activity and C) ALT, AST, and BUN activities in serum at 8 h after a single dose of free DOX and DOX‐loaded liposome at DOX dosage of 15 mg kg^−1^ with and without HBO therapy. Data as mean ± S.E. (*n* = 5). *P < 0.05, N.S. as not significant.

The most concerning hurdle preventing the use of the combination treatment of HBO with DOX is the side effects. As HBO therapy significantly increases oxygen nonselective toward the whole body, the amount of ROS is increased as well. If these ROS encounter free DOX, which randomly distribute within the entire body, severe adverse reactions will occur to endangered patients' lives. For this reason, simultaneous administration of DOX with HBO has been considered an absolute contraindication.[[qv: 8a]] One prominent advantage of Doxil is its attenuated side effects, particular for cardiotoxicity.^48^ To evaluate the cardiotoxicity of the combination therapy, the pathologic analysis on heart tissues harvested in antitumor experiment was conducted (**Figure** **6**A).

Cardiomyocytes in the control and HBO groups are arranged in a regular line with clear structure, indicating that no obvious cell damage occurred when HBO therapy was administered alone; this is consistent with the body weight changes we described above (Figure 5D). By contrast, for DOX and DOX+HBO, the nuclei of the immune cells gather together and lysis of the cytoplasm occurs due to the necrosis of cardiomyocytes, signifying cardiotoxicity. The damage to the cardiomyocytes in DOX+HBO is more obvious than that of DOX and further confirms that HBO does exasperate the cardiotoxicity of DOX. However, little histological damage is observed in Doxil and Doxil+HBO, suggesting cardiotoxicity is reduced for both groups. As the dosage of DOX used in the antitumor experiment is relatively low (4 mg kg^−1^), the pathologic analysis of heart tissue cannot fully characterize the side effects associated with DOX. To further investigate the safety of the combination therapy, 7 mg kg^−1^ DOX or equivalent dosage of Doxil was intravenously administered with or without HBO. Blood was collected 8 h after the injection and creatine phosphokinase (CK) activity, an indicator for damage of cardiomyocytes,^49^ was assayed with a Beckman Coulter AU5800 chemistry analyzer (Beckman Coulter, Kansas, US) (Figure 6B). Compared to the control (120 ± 12 U L^−1^) and HBO (117 ± 15 U L^−1^), serum CK activity increased in all DOX groups because of the cardiotoxicity caused by DOX. Treatment with DOX alone significantly increased the CK activity (170 ± 5 U L^−1^, *P* < 0.05) in serum. DOX combined with HBO therapy showed the highest CK activity (234 ± 48 U L^−1^) in serum, which justifies the absolute contraindication label. However, no significant differences were observed between the control and groups with Doxil (130 ± 16 U L^−1^ for Doxil and 126 ± 12 U L^−1^ for Doxil+HBO), revealing reduced cardiotoxicity of Doxil.

The toxicity of the combination therapy to other major organs was also evaluated. The activities of alanine aminotransferase (ALT) and aspartate aminotransferase (AST) were measured to evaluate the functions of liver, and blood urea nitrogen (BUN) was measured to evaluate the functions of kidney. Serum ALT, AST, and BUN levels decreased in all groups when Doxil was applied. However, no significant difference can be observed (Figure 6C). Pathological analysis showed in Figure S10 (Supporting Information) reveals that no abnormalities are observed in major organs after the combination treatment, including the heart, liver, spleen, lung, and kidneys.

One of the most important achievements of nanotherapeutics is the reduced side effects of antitumor agents. Zhang et al. uncovered that the key step for cardiotoxicity is the molecular binding between DOX and topoisomerase‐IIβ of cardiomyocytes.^50^ Application of Doxil reduces the chance of free DOX binding to topoisomerase‐IIβ and therefore reduces cardiotoxicity.[[qv: 11b,51]] Since the combination of DOX with HBO is considered an absolute contraindication, the application of Doxil can provide an alternative option for clinical translations. The CK activity in serum illustrates that no significant damage to cardiomyocytes occurs when Doxil is combined with HBO, again verifying that the combination of Doxil with HBO is safe.

Because HBO overcomes tumor hypoxia and decreases fibril deposition in tumor ECM, one may fear that HBO might induce or promote tumor metastasis. In this study, no metastasis of H22 tumor was observed at the end of the in vivo antitumor experiment. As H22 subcutaneous tumor is a relatively low metastatic tumor model, we investigated the effects of HBO on tumor metastasis on a 4T1 metastatic tumor model. After 10 HBO therapies, neither significant difference of lung weight nor number of metastatic nodules between the control and HBO was observed (Figure S11, Supporting Information). This suggests that there is no significant promotion of metastasis by HBO therapy. Our results are consistent with numerous previous studies, which all showed HBO did not promote tumor metastasis.^52^ In sum, HBO therapy does not promote tumor metastasis and is thus safe for in vivo application.

## 3 Conclusion

Two FDA approved therapies, HBO and Doxil, are rationally combined together for the first time to combat hypoxic solid tumors. By elevating oxygen tension, HBO not only promotes Doxil tumor penetration and accumulation via decreasing collagen deposition at the tumor ECM, but additionally sensitizes tumor cells to Doxil, further contributing to the enhanced antitumor efficacy. More importantly, the combination therapy does not show extra side effects that could prohibit the combination of HBO with conventional chemotherapies. The combination treatment also does not promote tumor metastasis. The results reported here clearly demonstrate that the simultaneous application of HBO and Doxil is a safe and acceptable combination. HBO, for example, potentiates Doxil but not free DOX, and improves tumor penetration and antitumor efficacy. In return, Doxil attenuates DOX‐induced adverse reactions under HBO, underlining the synergistic effects of combining HBO with Doxil. Our results further show that HBO is a relatively uncomplicated and effective therapy for tumor penetration and TME modulation. HBO can be combined with a wide variety of FDA approved nanomedicines against different hypoxic solid tumors.

In summary, the combination of HBO and Doxil has been proven to be a safe, effective, and robust therapy against hypoxic solid tumors. Given both therapies are approved by FDA and routinely applied in widespread clinics and practices, the combination of HBO with Doxil is a promising new modality for cancer chemotherapy and could easily be translated to clinical trials for patients with hypoxic solid tumors.

## 4 Experimental Section

Materials, details on HBO therapy, and procedures for combination therapies are included in the Supporting Information. All experimental procedures, the animal use, and care protocols were carried out under a protocol approved by the Animal Care and Use Committee of Huazhong University of Science and Technology.

## Conflict of Interest

The authors declare no conflict of interest.

## Supporting information

Supplementary
