# Supplementary material for: Hyperbaric Oxygen Potentiates Doxil Antitumor Efficacy by Promoting Tumor Penetration and Sensitizing Cancer Cells
Source: Adv Sci (Weinh). 2018 Jun 25;5(8):1700859. doi: 10.1002/advs.201700859 (PMC6097095; doi:10.1002/advs.201700859)
Supplement: Supplementary file 1 — Supplementary [file ADVS-5-1700859-s001.pdf]

Copyright WILEY-VCH Verlag GmbH & Co. KGaA, 69469 Weinheim, Germany, 2018.

ADVANCED SCIENCE
Open Access

Supporting Information

for *Adv. Sci.,* DOI: 10.1002/advs.201700859

Hyperbaric Oxygen Potentiates Doxil Antitumor Efficacy by Promoting Tumor Penetration and Sensitizing Cancer Cells

Xian Wu, Yanhong Zhu, Wei Huang, Jingqiu Li, Bixiang Zhang, Zifu Li, and Xiangliang Yang**

Supporting Information

Hyperbaric Oxygen Potentiates Doxil Antitumor Efficacy by Promoting Tumor Penetration and Sensitizing Cancer Cells

Xian Wu, *Yanhong Zhu*, *Wei Huang*, *Jingqiu Li*, *Bixiang Zhang*, *Zifu Li**, and *Xiangliang Yang**

Xian Wu, Prof. Yanhong Zhu, Wei Huang, Jingqiu Li, Prof. Zifu Li, Prof. Xiangliang Yang

National Engineering Research Center for Nanomedicine, College of Life Science and Technology, Huazhong University of Science and Technology, Wuhan 430074, P. R. China

E-mail: zifuli@hust.edu.cn; yangxl@hust.edu.cn

Prof. Bixiang Zhang

Huazhong University of Science and Technology, Tongji Med College, Tongji Hospital, Hepat Surg Ctr, 1095 Jiefang Ave, Wuhan 430030, P. R. China

Prof. Zifu Li

Wuhan Institute of Biotechnology, High Tech Road 666, East Lake High Tech Zone, Wuhan, 430040, P. R. China

Xian Wu and Prof. Yanhong Zhu contributed equally to this work.

Experimental section

Materials

The experimental hyperbaric oxygen (HBO) animal chamber was purchased from Weifang Huaxin Oxygen Industry Co., Ltd (Weifang, China). The three-gas incubator was purchased from Changsha Huaxi Electronic Technology Co., Ltd (Changsha, China). Doxorubicin hydrochloride was bought from Beijing HuaFeng United Technology Co., Ltd. (Beijing, China). Doxorubicin-loaded liposome (Li Bao Duo®) was obtained from Wuhan General Hospital of Guangzhou Military (Wuhan, China). All other chemicals used were of analytical grade and commercially available.

In vitro and *in vivo* experiments were carried out using mouse hepatocellular carcinoma cell line H22, human hepatocellular carcinoma cell line Bel-7402, and mouse breast cancer cell line 4T1 [China Center for Type Culture Collection (CCTCC)]. Cells were cultured with RPMI-1640 cell culture medium (Thermo) supplemented with 10% FBS (Gibco) at 37°C with 5% CO_2. Balb/c mice (male, 18-20 g) were purchased from Hubei Center for Disease Control and Prevention (Hubei Provincial Laboratory, Animal Qualification Certificate No. 4200695200). Balb/c-nude mice (male, 14-16 g) were purchased from Beijing Wei Tong Li Hua experimental animal Co., Ltd (Beijing, China). Mice were housed in a specific-pathogen-free animal room with controlled temperature (25 ± 0.2°C). All animal studies were approved by the Animal Experimentation Ethics Committee of Huazhong University of Science and Technology, and carried out in compliance with guidelines approved by the Science and Technology Department of

Hubei Province.

HBO protocol

The experimental HBO chamber was sterilized with 75% ethanol. Cages of each mouse group were then moved into the chamber. The chamber was ventilated with 100% oxygen for one minute and the door of the chamber was then closed. HBO therapy was administrated at a pressure of 2.5 ATM for 90 minutes. Fifteen minutes of pressurization and 15 minutes of depressurization was allowed for the mice to adjust to the change of pressure. The oxygen content in the chamber was monitored with an oximeter. To maintain >97% oxygen atmosphere at all times, the chamber was flushed with pure oxygen for 3–5 minutes every 10–30 minutes depending on the number of animals in the chamber.

Tumor models

For the H22 subcutaneous tumor model, 0.1mL of H22 cells (2×10^{7} cells mL^{-1}) in saline solution were injected into the right flank of male Balb/c mice. For the Bel-7402 subcutaneous tumor model, 0.1mL of Bel-7402 cells (3×10^{7} cells mL^{-1}) in saline solution were injected into the right flank of male Balb/c-nude mice. For the 4T1 metastatic tumor model, 0.1mL of 4T1 cells (1×10^{7} cells mL^{-1}) in saline solution were injected into the right flank of female Balb/c mice.

Detection of tumor hypoxia change

To detect variations of tumor hypoxia after HBO therapy, a hypoxia-probe, immunohistochemistry, and western blot was applied. The Hypoxyprobe-1™ Kit

(Hypoxyprobe Inc, Burlington, MA, US) was used for detection of a hypoxic area within the tumor tissue. Briefly, after mice received a single dose of HBO therapy for two hours, pimonidazole (dissolved in saline) was intravenously injected through the tail vein at a dosage of 60 mg kg^{-1}. For the control group, no HBO therapy was conducted. Ninety minutes after injection, tumor tissue was harvested and fixed. Anti-pimonidazole mouse IgG_1 monoclonal antibody (MAb1) was then used for immunochemical detection of hypoxic area in the tumor tissue.

Immunohistochemistry analysis of HIF-1α and VEGF was also applied to detect changes of hypoxia in the tumor tissue. After HBO therapy, tumors were immediately fixed and the standard protocol of immunohistochemistry was performed. Mouse monoclonal HIF-1α antibody (Santa, catalog number Sc-53546) and mouse monoclonal VEGF antibody (Santa, catalog number Sc-7269) were used for staining HIF-1α and VEGF, respectively.

The expression of HIF-1α was quantified by a western blot experiment. After HBO therapy, the total protein of the tumor tissue was extracted. Antibody against HIF-1α was obtained from BD Biosciences (Franklin Lakes, NJ, USA). Antibody against β-Actin was purchased from Santa Cruz Biotechnology (Santa Cruz, CA, USA). Standard protocol of western blot was followed and proteins were visualized with an ImageQuant LAS 4000 mini system (GE Healthcare, Piscataway, NJ, USA) according to the manufacturer's instructions.

The effect of HBO on tumor ECM

To verify that HBO therapy could modulate tumor ECM, Masson's trichrome staining was applied to measure the content of fibril content in the tumor tissue. The transcription and expression of CTGF and collagen I were also quantified. H22 tumor-bearing mice were separated into two groups. Mice in the control group were kept under normal conditions, whereas mice in HBO group received HBO therapy every day for 3 days. At the end of the last therapy, mice were sacrificed and tumor tissues were collected and fixed. For the Masson's trichrome staining analysis, standard protocol was applied

For the qRT-PCR analysis, total tumor tissue RNA was extracted with TRIzol reagents (Life Technologies), according to the manufacturer's instructions; the primer sequences used are shown as Table S1. Subsequently, 1 μg of RNA was used for reverse transcription using TransScript First-Strand cDNA Synthesis SuperMix (TransGen). Afterwards, 1 μL of cDNA was amplified with GoTaq qPCR Master Mix (Promega) in triplicate. The expression of mRNA was quantified by a CFX Connect Real-Time System (BIO-RAD).

For the western blotting analysis, the total protein of tumor tissue was extracted. A standard western blotting protocol was followed with the appropriate antibodies. Antibodies against collage I and CTGF were obtained from Abcam (Shanghai, China). And the antibody against β-Actin was purchased from Santa Cruz Biotechnology (Santa Cruz, CA, USA). Proteins were visualized with an ImageQuant LAS 4000 mini system (GE Healthcare, Piscataway, NJ, USA) according to the manufacturer's instructions.

Measurement of in vivo tumor penetration and DOX concentration

To measure the penetration and accumulation of DOX in the tumor tissue after HBO therapy, 20 tumor-bearing mice were randomly separated into 4 groups (n=5): DOX, Doxil, DOX+HBO, and Doxil+HBO. Mice in the two HBO related groups (DOX+HBO and Doxil+HBO) were pre-treated with 2 HBO therapies (once a day for 2 days). Mice in the DOX and DOX+HBO groups received one administration of DOX dissolved in saline at a dose of 7 mg kg^{-1} (0.2 mL) via intravenous injection. Similarly, mice in the Doxil and Doxil+HBO groups were injected with Doxil at the equivalent dose of 7 mg kg^{-1} DOX. Another single HBO therapy was immediately conducted after the injection of DOX or Doxil. Twenty-four hours after injection, mice were sacrificed and the tumor tissues were collected.

Tumor penetration measurement

To measure the penetration depth of DOX, the cryotomy of the tumor tissues were first obtained. The frozen tumor sections were stained with a FITC-CD31 antibody (Abcam, Cambridge, UK). The fluorescence of tumor slides was observed under an Olympus FV1000 confocal microscope.

Penetration distance between the Drugs and Vessels

The distance between the DOX and vessels was calculated with simulated scatter diagrams [1]. Within these diagrams, the green points represent the vessel, the red points represent the DOX, and the yellow points represent the colocation of the vessel and DOX, Figures 3A and S4. Using a Rectangular Plane Coordinate System, the X- and Y-coordinates of each point is recorded. Based on these coordinates, the minimum

distance between every red point and the nearest green point is calculated using the following formula:

$$D = \sqrt{(X_{red} - X_{green})^2 + (Y_{red} - Y_{green})^2}$$

After counting the red points from several fluorescence images of each sample, the average value was taken as the distance between the drugs and the vessels.

Quantification of DOX concentration in tumor

To quantify DOX concentration in tumor tissue, tumors were rinsed with saline, dried and weighted. Tumor tissue (0.05 g) was homogenized using a glass homogenizer with 1mL PBS in an ice bath. After homogenization, 1 mL of methanol/HCl (94/6, v/v) was added and the mixtures were vortexed for 5 minutes at room temperature. Fifteen minutes was given to allow tissues to excrete DOX, afterwards, the mixtures were centrifuged (10000 rpm) at 4°C for 20 minutes to precipitate proteins. Following this, 100 µL of supernatant was analyzed using Flexstation3® microplate reader (Molecular Device, CA, US) with excitation and emission wavelengths set at 480 and 590 nm, respectively. The concentration of DOX (mg g^{-1} tissue) in each tumors were calculated with a pre-measured calibration curve.

Analysis of HBO's effect on cell cycle arrest, internalization and cytotoxicity of doxorubicin

Cell cycle measurement

To investigate the effects of HBO therapy on cell cycle both *in vitro* and *in vivo*, Bel-7402 cell line and Bel-7402 subcutaneous tumor model were used. To measure changes on cell

cycle *in vitro*, Bel-7402 cells were cultured in 6-well plates at 1 × 10^6 cells per well. After incubation for 24 hours, cells were divided into 5 groups to be given different treatment (Table S2). After different therapies, cells were washed with cold PBS 3 times and then fixed overnight with 70% ethanol under 4°C. Cells were washed again with cold PBS and incubated with 500 μL working solution (50 μg mL^{-1} PI and 100 μg mL^{-1} RNase A in PBS) under 4°C for 30 minutes. Flow cytometry was then applied for PI detection. Results were analyzed using the CXP analysis software (Beckman Coulter, Kansas, US).

To detect changes on cell cycle *in vivo*, nude mice bearing Bel-7402 subcutaneous tumor were randomly separated into 2 groups: mice housed under normal condition for 5 days (controls) and mice that received 1 HBO therapy per day for 5 days (HBO-treated). Twenty-four hours after the last treatment, mice were sacrificed and the tumors were harvested and washed with cold PBS 3 times. Tumor tissues were cut into small pieces in FBS-free cell culture with collagenase for 90 minutes. Crumbled small pieces of tumor tissues were applied to frosted glass slides to isolate single tumor cells. The obtained cell suspension was then forced through a 200-mesh sieve, washed with cold PBS for 3 times and then fixed with 70% ethanol under 4°C overnight. Cells were washed again with cold PBS and incubated with 500 μL working solution (50 μg mL^{-1} PI and 100 μg mL^{-1} RNase A in PBS) under 4°C for 30 minutes. Flow cytometry was then applied for PI detection. Results were analyzed using the CXP analysis software (Beckman Coulter, Kansas, US).

Cellular uptake and efflux measurement

To analyze cellular uptake and efflux of DOX under different conditions, H22 cells were

cultured in 12-well plates at a density of 3 × 10^5 cells per well. After incubation for 24 hours under either normoxia (21% O_2) or hypoxia (1% O_2), 5 µg $mL^{-1}$of DOX was added to each well. Cells were washed with PBS 3 times after 2 hours of incubation and the fluorescence of DOX in cells was measured immediately with flow cytometry using an FC500 cytometer (Beckman Coulter, Kansas , US). The results were analyzed using the CXP analysis software (Beckman Coulter, Kansas, US). The fluorescent intensity of DOX was recorded as the initial content of DOX in cells. The cells were then incubated for another 4 hours under either normoxic or hypoxic conditions. After washing with PBS 3 times, the fluorescent intensity in cells was measured again and recorded as the final content of DOX in cells. The difference between the initial and the final content of DOX represents the efflux content of DOX.

IC50 of DOX under normoxia and hypoxia

To investigate the cell-killing effect of DOX under different conditions, cell viability was measured with the Cell Counting Kit-8 (CCK-8) assay kit (Dojindo Corp.). Ten thousand H22 cells were plated into each well of a 96-well plate with 100 µL of culture medium. The appropriate concentration of DOX was added using the serial dilution method. The cells were subsequently incubated at 37°C under either normoxia (21% O_2) or hypoxia (1% O_2) for 24 hours or 48 hours. 10 µL CCK-8 was added to each well. After incubating for 4 hours, the cell density was measured indirectly through quantification of the solubilized formazan product at 450 nm with Flexstation3® microplate reader (Molecular Device, CA, US). Three independent experiments were performed.

In vivo antitumor activity of combination therapies

To test the antitumor activity of combination therapies *in vivo*, H22 subcutaneous tumor-bearing mice were randomly separated into 6 groups (n=5): Control, HBO, DOX, Doxil, DOX +HBO and Doxil+HBO (Table S4). Body weight and tumor size of each mouse were measured every other day. At the end of the last therapy, all mice were sacrificed. The tumor weight and tumor volume were measured. Tumor and heart tissues were collected for histological analysis. The tumor size was determined with a caliper in two dimensions. The tumor volume and the tumor growth inhibition rate (according to tumor weight) were calculated.

Side effects evaluation

To evaluate the damage of combination therapies to the heart, heart tissues collected from the pharmacodynamics study were fixed and standard H&E staining protocol was conducted for histological analysis. To further analyze the side effect of different therapies, serum biochemical analysis was carried out. Mice were randomly divided into 6 groups: control, HBO, DOX, DOX+HBO, Doxil, Doxil+HBO. A 7 mg kg^{-1} DOX or equivalent dosage of Doxil was intravenously administered with or without HBO. Eight hours after injection, serum was collected. The activity of creatine phosphokinase (CK), alanine aminotransferase (ALT), aspartate transaminase (AST) and blood urea nitrogen (BUN) were analyzed by Beckman Coulter AU5800 chemistry analyzer (Beckman Coulter, Kansas , US).

HBO's effect on tumor metastasis

To investigate the influence of HBO therapy on tumor metastasis, a 4T1 metastatic tumor model was used. Ten days after inoculation, tumor-bearing mice were randomly separated into 2 groups: the control group and the HBO-treated group. Mice in the HBO-treated group received HBO therapy every other day for 10 times, whereas mice in the control group were housed under normal conditions. After 10 HBO therapies, mice in both groups were sacrificed, and lungs were collected and weighted. Lung tissues were then fixed in Bouin's solution for 3 hours and washed in 95% ethanol overnight. The metastatic nodules were counted.

Statistical analysis

The data were expressed as mean ± SE. Statistical significance in all experiment was determined using one-way ANOVA followed by a Student's test for multiple comparison tests. Statistical analysis was analyzed using statistical software (SPSS 17.0, Chicago, USA).

Supplementary Figures

For characterization of hydrodynamic diameter and zeta potential of Doxil before and after HBO treatment, Doxil was first dissolved in double distilled water and the diameter and zeta potential were then tested with a Nano ZS90 (Marlven Instrument, UK).

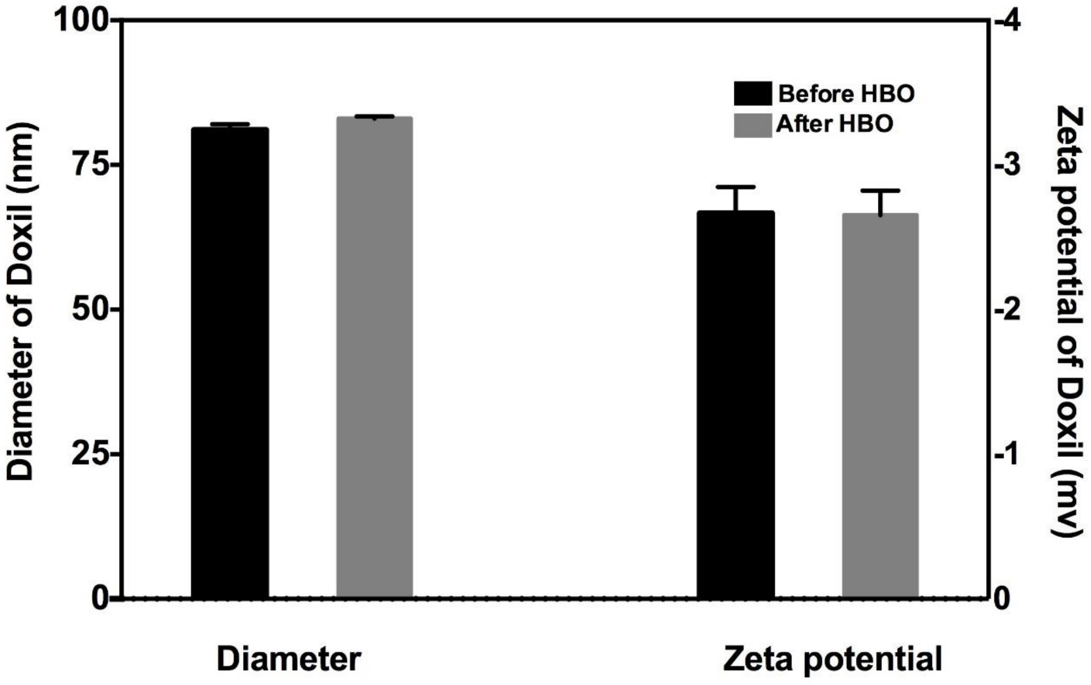

Figure S1. Diameter and zeta potential of Doxil before and after HBO treatment. Data as mean ± S.E. (n=3).

For our drug release experiment, dialysis was used. A concentration of 0.1 mL of Doxil (contain 100μg DOX) was added into the dialysis bag. Twenty-five mL of PBS (pH7.4) was used as the release medium. The shaker speed was 140 rpm and the temperature was set as 37℃. Samples were collected at the indicated time points and the DOX spectrum was measured with fluorescence spectrophotometry (Ex=488 nm, Em=556 nm); the concentration was calculated with a pre-measured calibration curve.

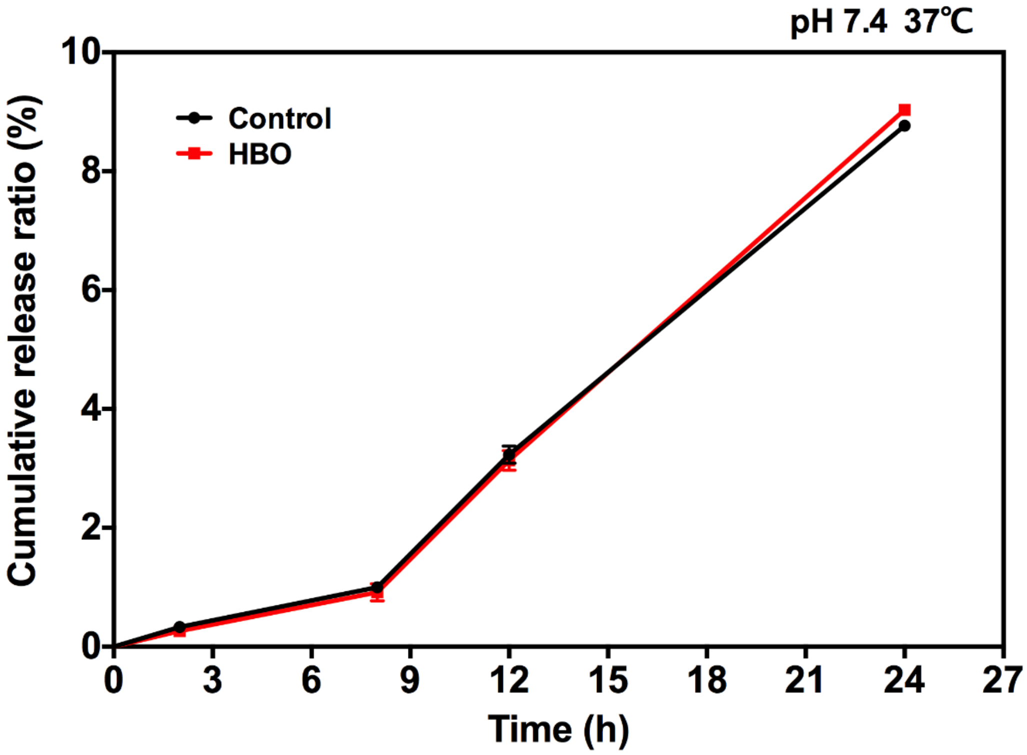

Figure S2. *In vitro* release profile of Doxil in PBS (pH 7.4) before and after HBO treatment. Data as mean ± S.E. (n=3).

For the immunohistochemistry analysis of VEGF, tumors were immediately fixed after 2 hours of HBO treatment. The standard protocol of immunohistochemistry was performed using mouse monoclonal VEGF antibody (Santa, catalog number Sc-7269).

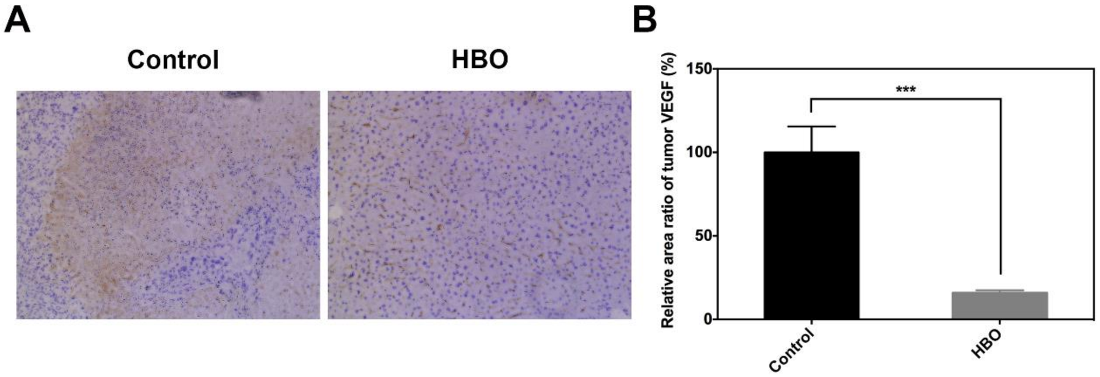

Figure S3. Immunohistochemistry analysis of VEGF (A) and the semi-quantification (B). Data as mean ± S.E. (n=3). ***P<0.001

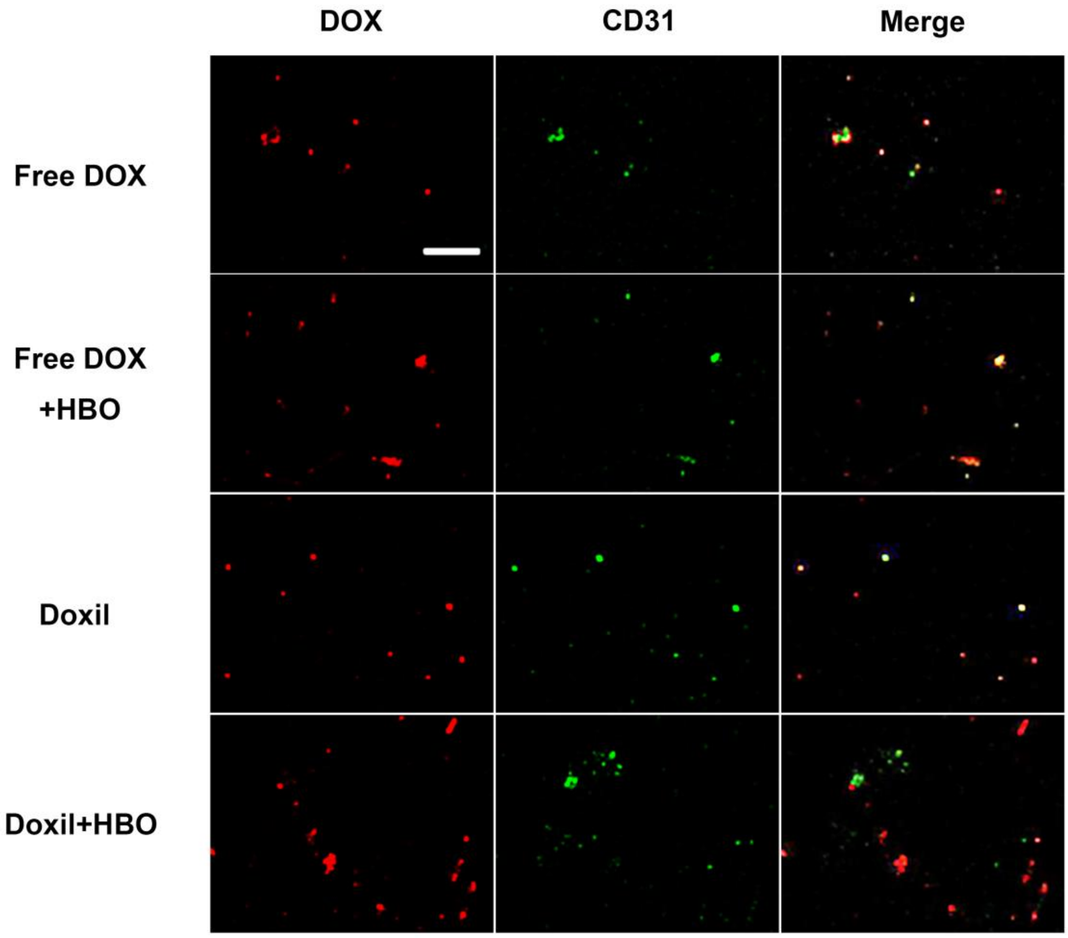

Figure S4. Zoom-in pictures of *in vivo* penetration of DOX into the tumors of H22-bearing mice after intravenous injection of free DOX/ Doxil at DOX dosage of 7 mg kg-1 with and without HBO therapy. The frozen tumor sections were observed at 24 hours after injection using confocal microscopy. The blood vessels were stained by FITC-CD31 antibody. The scale bar is 200 μm for all images.

For DOX biodistribution after different treatments, mice were divided into four groups: DOX (4mg kg^{-1} DOX, intravenous injection), DOX+HBO (4mg kg^{-1} DOX, intravenous injection, immediately followed with 1 HBO treatment), Doxil (equivalent dosage of 4mg kg^{-1} DOX, intravenous injection) and Doxil+HBO (equivalent dosage of 4mg kg^{-1} DOX, intravenous injection, immediately followed with 1 HBO treatment). Two, four and eight hours after injection, mice were sacrificed and tissues were harvested. Tissues were rinsed with saline water, dried, and weighted. Tissues (0.05 g) were homogenized using a glass homogenizer with 1 mL PBS in an ice bath. After homogenization, 1 mL of methanol/HCl (94/6, v/v) was added and the mixtures were vortexed for 5 minutes at room temperature. After allowing 15 minutes for tissues to excrete DOX, the mixtures were centrifuged (10000 rpm) at 4°C for 20 minutes to precipitate proteins. Then 100 µL of supernatant was analyzed using Flexstation3® microplate reader (Molecular Device, CA, US) with excitation and emission wavelengths of 488 and 556 nm, respectively. The concentrations of DOX (µg g^{-1} tissue) in tissues were calculated with a pre-measured calibration curve.

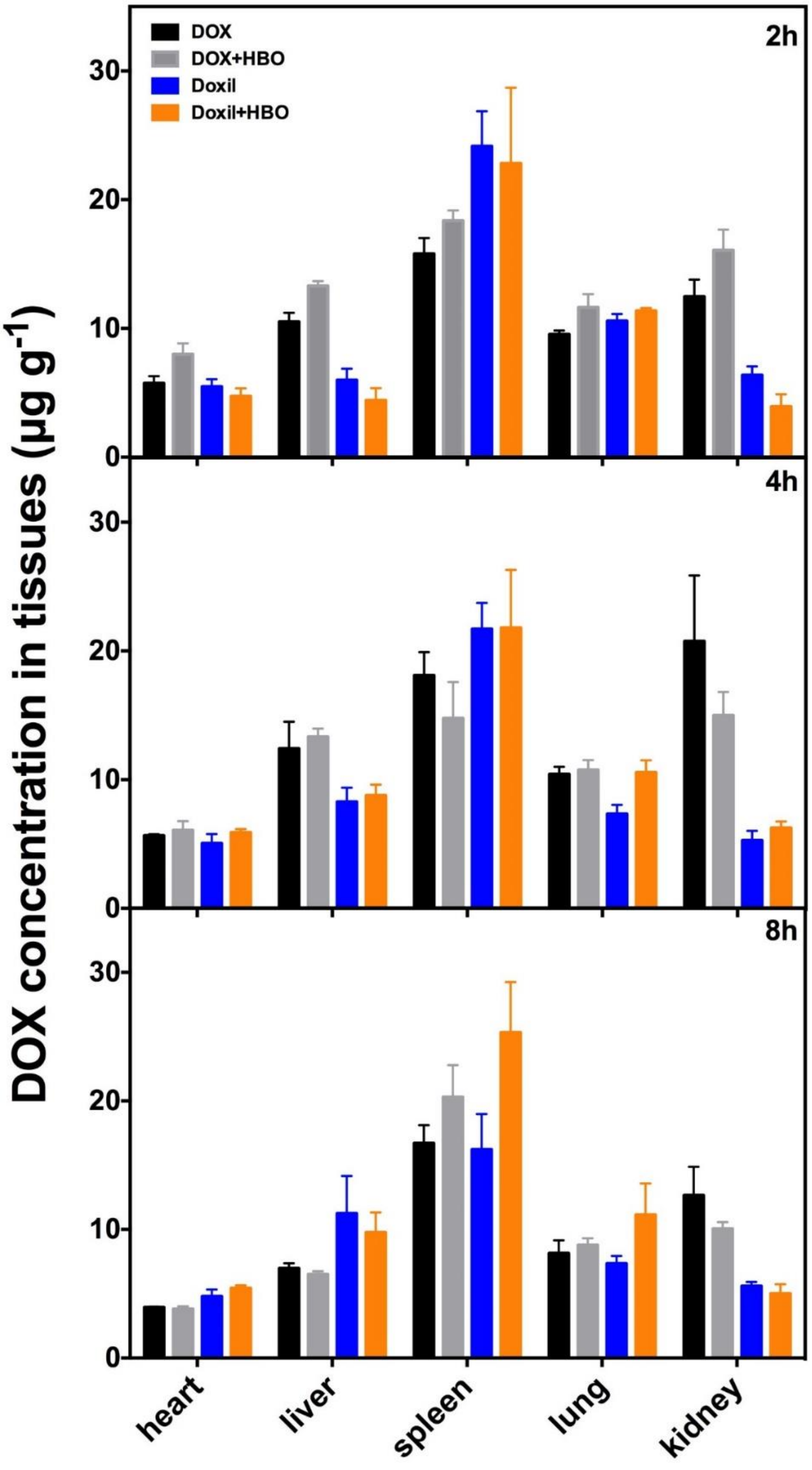

Figure S5. Biodistribution of DOX in different groups after 2, 4, and 8 hours. Data as mean ± S.E. (n=5).

To measure the effects on the cell cycle in the tumor tissue, Bel-7402 tumor-bearing mice were randomly divided into two groups: control and HBO-treated. Mice in the HBO-treated group were given HBO therapy daily for 5 days. 24 hours after the last therapy, tumor tissue was harvested and washed in cold PBS for 3 times. Tumor tissue was subsequently cut into small pieces and placed in a FBS-free cell culture with collagenase for 90 minutes. Next, crumbled small pieces of tumor tissues were applied to frosted glass slides to isolated single tumor cells. The obtained cell suspension was forced through a 200-mesh sieve, washed with cold PBS 3 times and fixed overnight with 70% ethanol under 4°C. Cells were washed again with cold PBS and incubated with 500 μL working solution (50 μg mL^{-1} PI and 100 μg mL^{-1} RNase A in PBS) under 4°C for 30 minutes. Flow cytometry was then applied for PI detection. Results were analyzed using the CXP analysis software (Beckman Coulter, Kansas, US).

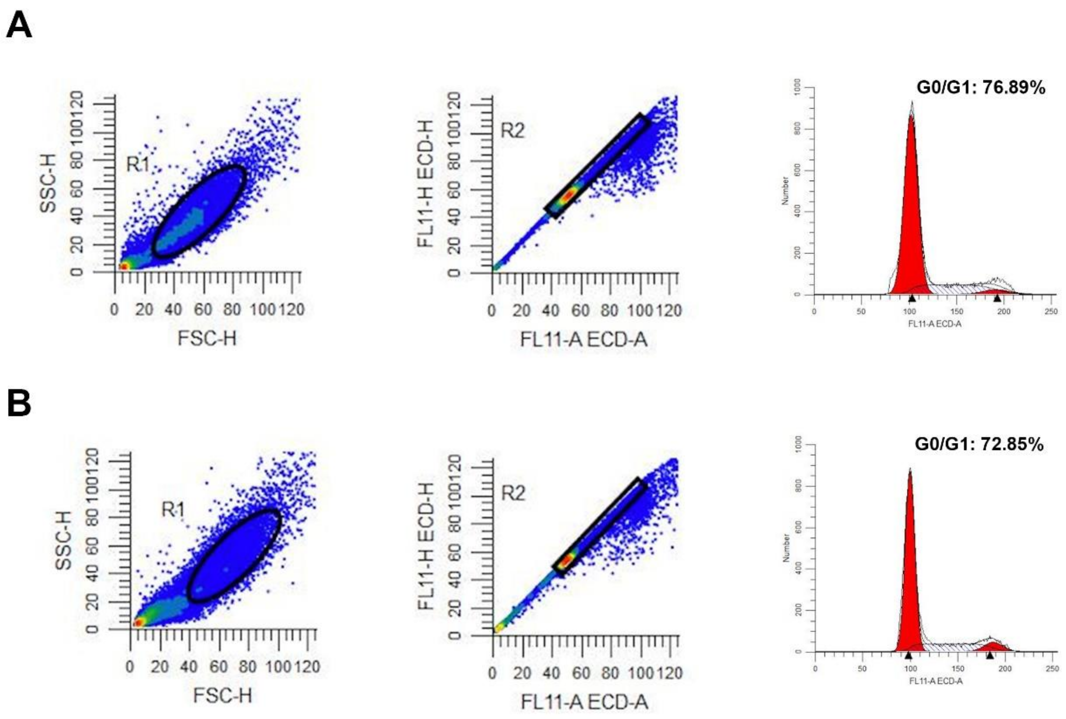

Figure S6. Representative raw data of cell cycle measurement in tumor tissue under normal condition (A) and HBO condition (B).

To measure the effects on the cell cycle of Bel-7402 cells in vitro, cells were divided into 5 groups: control, Doxil, Hypoxia, Hypoxia+Doxil and Hypoxia+Doxil+HBO (see Table S2). After treatments, cells were washed with cold PBS and fixed overnight with 70% ethanol. For PI staining, cells were incubated with 500 μL working solution (50 μg mL^{-1} PI and 100 μg mL^{-1} RNase A in PBS) under 4°C for 30 minutes. Flow cytometry was then applied for PI detection. Results were analyzed using the CXP analysis software (Beckman Coulter, Kansas, US).

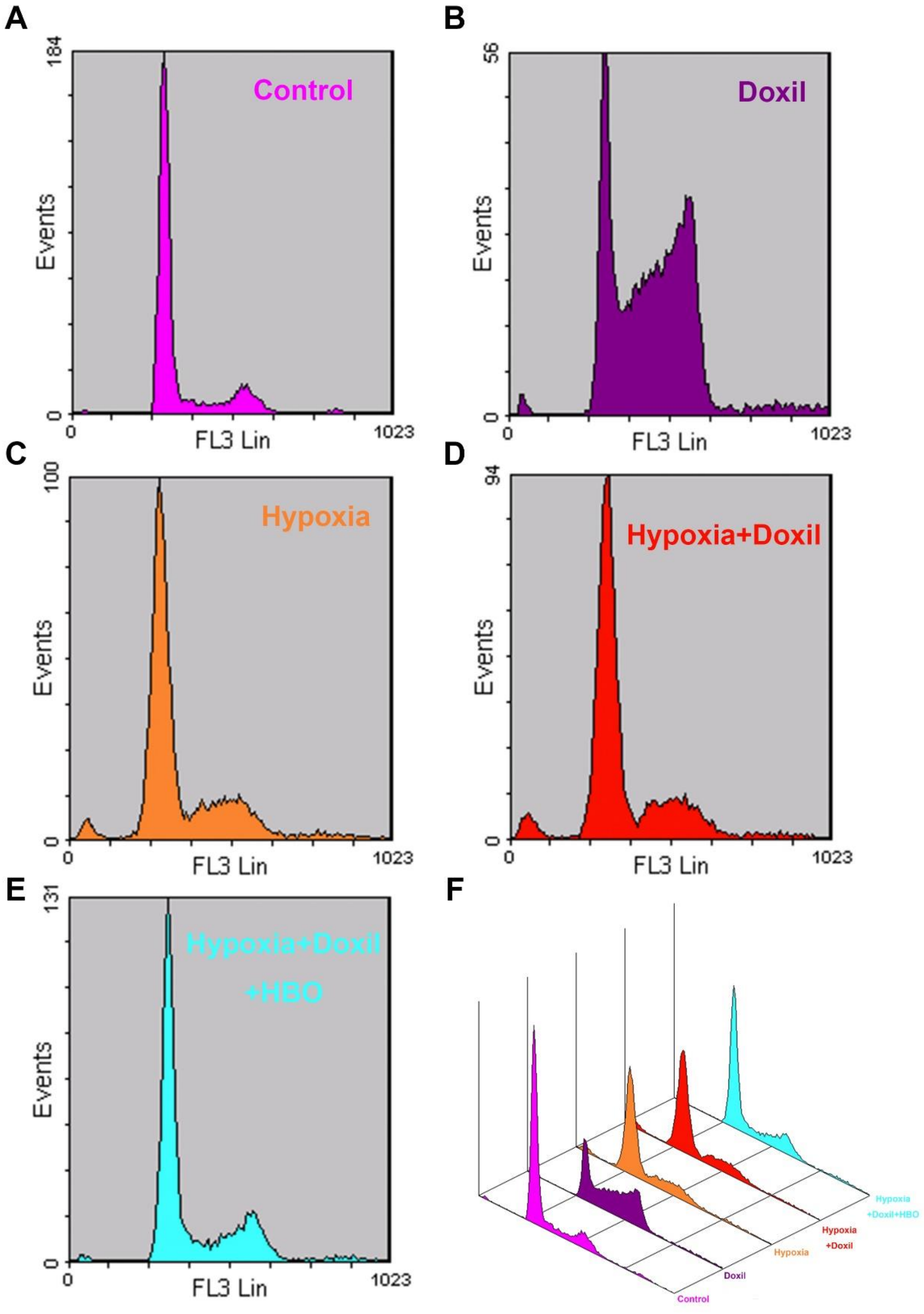

Figure S7. Representative raw data of cell cycle measurement of Bel-7402 cell *in vitro* under different treatments: Control (A), Doxil (B), Hypoxia (C), Hypoxia+Doxil (D) and Hypoxia+Doxil+HBO (E). (F) Merged results of (A)-(E).

To detect cell viability after 24 hours of incubation with DOX under either normoxic and hypoxic conditions, 10,000 H22 cells were plated into each well of a 96-well plate with 100 µL of culture medium. The appropriate concentration of DOX was added using the serial dilution method. The cells were subsequently incubated at 37°C under either normoxia (21% O_2) or hypoxia (1% O_2) for 24 hours. 10 µL CCK-8 was add to each well. After incubating for 4 hours, cell density was measured using the solubilized formazan product at 450 nm with Flexstation3® microplate reader (Molecular Device, CA, US). Three independent experiments were performed.

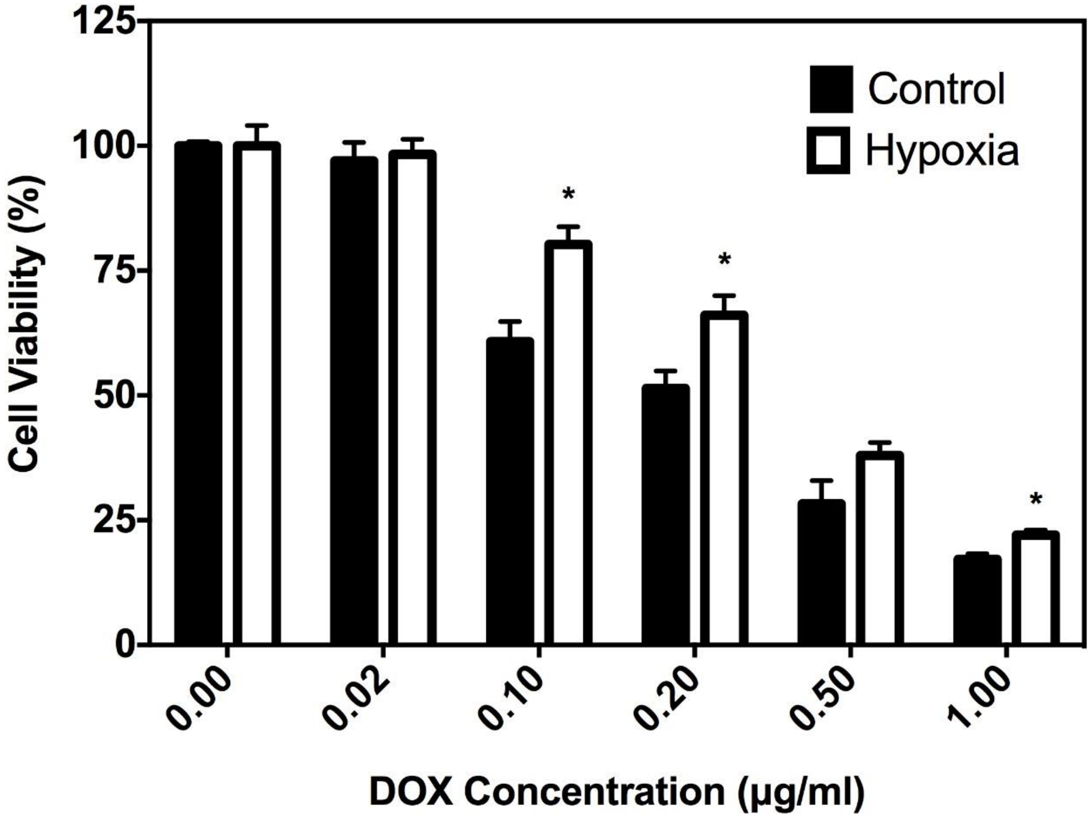

Figure S8. The cell viability of H22 cells treated with different concentrations of DOX at 24 hours as measured by CCK-8 assay. Data as mean ± S.E. (n=5). *P<0.05.

To assess whether HBO therapy alone has any cell-killing ability, different cells were plated into wells of a 96-well plate with 100 µL of culture medium. When 50% confluence was reached, cells were treated with a single HBO treatment daily for 5 days; cells were returned to normal incubation condition after HBO treatment. At the end of the last treatment, 10 µL of CCK-8 was added to each well. After incubating for 4 hours, the cell density was measured with solubilized formazan product at 450 nm with Flexstation3® microplate reader (Molecular Device, CA, US). Three independent experiments were performed.

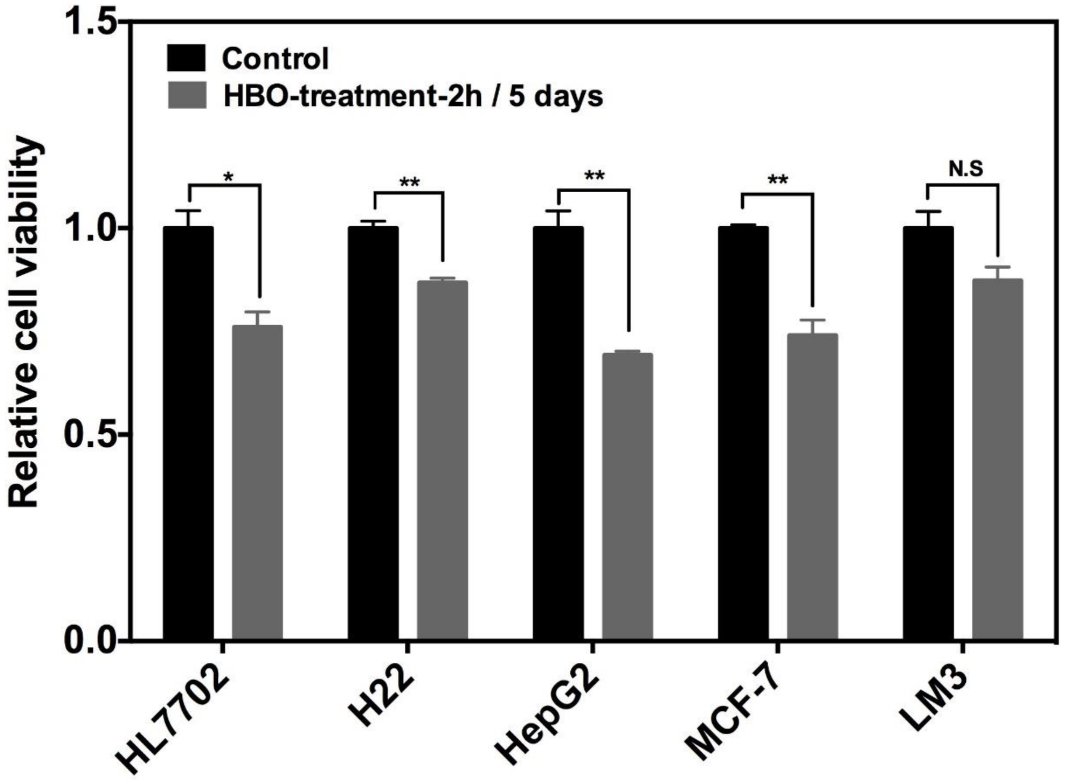

Figure S9. Relative cell viability of different cell lines after treated with HBO treatment 2 hours per day for 5 days. Data as mean ± S.E. (n=5). *P<0.05, **P<0.05. N.S. represents not significant.

To test whether the combination therapy has any side effect on major organs, H&E analysis of mouse organs was performed after 6 different treatments: control, HBO alone, 15 mg kg^{-1} DOX or equivalent dosage of Doxil with and without HBO. At the end of different treatments, mice were sacrificed and organs, including the heart, liver, spleen, lung and kidney, were harvested and fixed. Standard protocol of H&E analysis was then carried out.

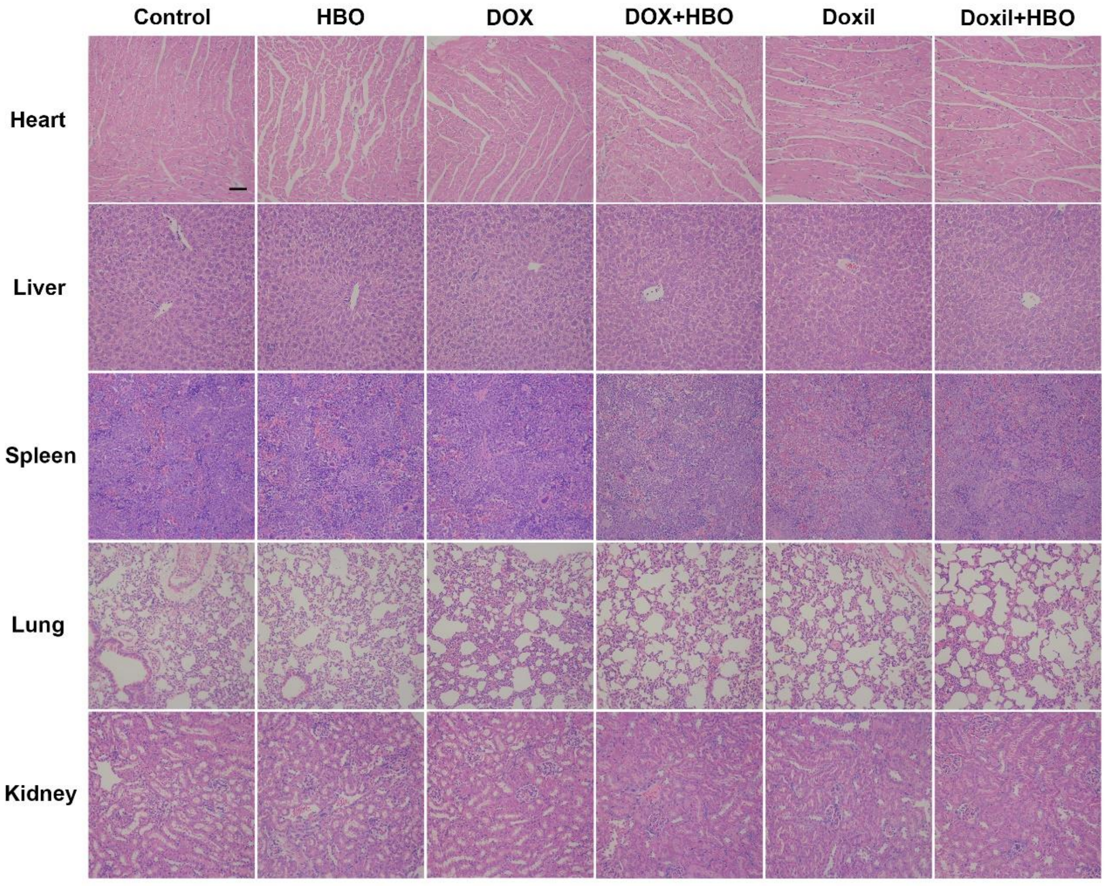

Figure S10. H&E analysis of major organs after mice receiving different treatments. The scale bar is 20 μm for all pictures.

To study the influence of HBO therapy on tumor metastasis, 4T1 subcutaneous

bearing mice received 10 HBO treatments during 20 days, one treatment every two days (Figure S11A). At the end of the experiment, the mice were sacrificed and the lungs were harvested. Lung tissues were then washed with saline and fixed with Bouin's solution for 30 minutes. Tissues were then washed overnight with 70% ethanol. The number of metastatic nodules was counted and the mass of the lung tissues was recorded.

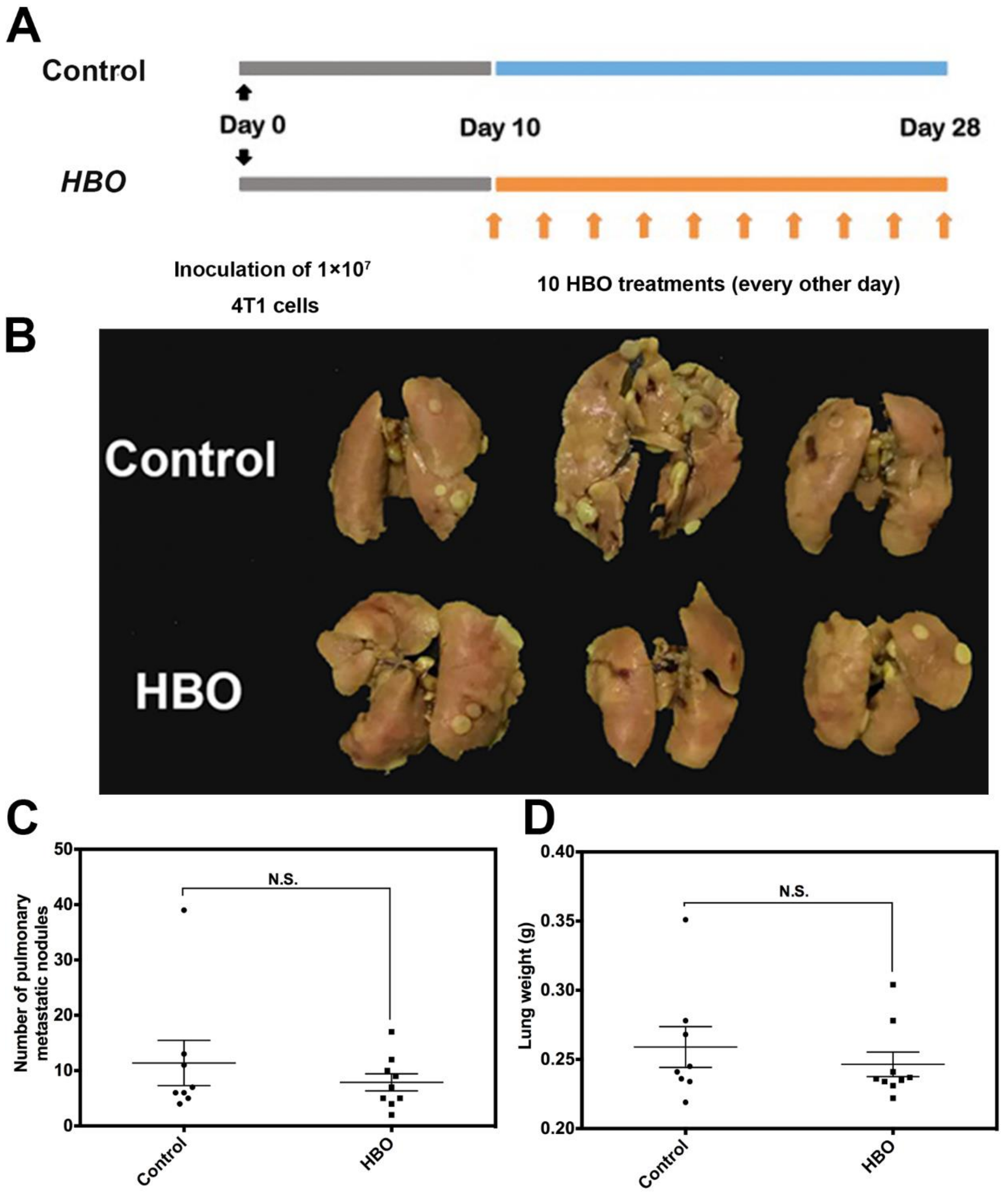

Figure S11. The effect of HBO on tumor metastasis with a 4T1 tumor model. (A) Brief schematic of the experimental procedures. (B) Representative pictures of lung tissues after treatment, the yellow dots represent metastatic nodules. (C) Lung weight after treatment. (D) The number of pulmonary metastatic nodules. Data as mean ± S.E. (n=9). N.S. represents not significant.

Table S1. Primer sequences used for qRT-PCR

Primer	Direction	Sequence
mus-Gadph	F	AGAAGGTGGTGAAGCAGGCATC
	R	CGGCATCGAAGGTGGAAGAGT
mus-CTGF	F	AGCTGCCTACCGACTGGAAG
	R	AGATGCCCATCCCACAGGTC
mus-Collagen	F	CCCGCCGATGTCGCTAT
	R	GCTACGCTGTTCTTGCAGTGAT

Table S2. Group sets for *in vitro* cell cycle experiment

Group	Treatment
Control	SC[a] for 48 h
Doxil	SC for 24h and incubated with Doxil[b] under SC for 24 h
Hypoxia	HC[c] for 48 h
Hypoxia+Doxil	HC for 24 h and incubated with Doxil under HC for 24 h
Hypoxia+Doxil+HBO	HC for 24 h and incubated with Doxil immediately
	Followed with 2 h HBO treatment and then under HC for 22 h

a: SC for standard cell culture condition (DMEM medium+10%FBS, 37℃, 21%O_2, 5%CO_2)

b: Doxil at dosage of 6 μg mL^{-1} of DOX

c: HC for hypoxic cell culture condition (DMEM medium+10%FBS, 37℃, 1%O_2, 5%CO_2)

Table S3. Detailed statistics of *in vitro* cell cycle measurement.

	G0/G1 (%)	S (%)	G2/M (%)
Control	51.7	27.9	20.4
Doxil	22.9[a,b,c]	51.6	25.5
Hypoxia	71.9[a]	19.5	8.6
Hypoxia+Doxil	73.5[a]	18.3	8.2
Hypoxia+Doxil+HBO	66.1[a,b,c]	20.5	13.4

a: Significant difference when compared with Control.

b: Significant difference when compared with Hypoxia.

c: Significant difference when compared with Hypoxia+Doxil

Table S4. Group sets for in vivo anti-tumor evaluation

Group	Treatment[a]
Control	0.2 mL saline (*i.v.)*
HBO	0.2 mL saline (*i.v.)* immediately *followed with HBO treatment*
DOX	0.2 mL DOX[b] (*i.v.)*
DOX+HBO	0.2 mL DOX[b] (*i.v.)* immediately *followed with HBO treatment*
Doxil	0.2 mL (*i.v.)* Doxil[c]
Doxil+HBO	0.2 mL Doxil[c] (*i.v.)* immediately *followed with HBO treatment*

a: Treatment was carried out every other day for 3 times

b: DOX was dissolved in 0.2 mL saline at a dosage of 4mg kg^{-1} body weight

c: Doxil was dissolved in 0.2 mL saline at an equivalent DOX dosage of 4mg kg^{-1} body weight

Reference

[1] T. Wei, J. Liu, H. L. Ma, Q. Cheng, Y. Y. Huang, J. Zhao, S. D. Huo, X. D. Xue, Z. C. Liang, X. J. Liang, *Nano Lett.* **2013**, 13, 2528.
